# Unique Responsiveness of Angiosperm Stomata to Elevated CO_2_ Explained by Calcium Signalling

**DOI:** 10.1371/journal.pone.0082057

**Published:** 2013-11-20

**Authors:** Timothy J. Brodribb, Scott A. M. McAdam

**Affiliations:** School of Plant Science, University of Tasmania, Hobart, Tasmania, Australia; University of Washington, United States of America

## Abstract

Angiosperm and conifer tree species respond differently when exposed to elevated CO_2,_ with angiosperms found to dynamically reduce water loss while conifers appear insensitive. Such distinct responses are likely to affect competition between these tree groups as atmospheric CO_2_ concentration rises. Seeking the mechanism behind this globally important phenomenon we targeted the Ca^2+^-dependent signalling pathway, a mediator of stomatal closure in response to elevated CO_2_, as a possible explanation for the differentiation of stomatal behaviours. Sampling across the diversity of vascular plants including lycophytes, ferns, gymnosperms and angiosperms we show that only angiosperms possess the stomatal behaviour and prerequisite genetic coding, linked to Ca^2+^-dependent stomatal signalling. We conclude that the evolution of Ca^2+^-dependent stomatal signalling gives angiosperms adaptive benefits in terms of highly efficient water use, but that stomatal sensitivity to high CO_2_ may penalise angiosperm productivity relative to other plant groups in the current era of soaring atmospheric CO_2_.

## Introduction

Land plants rely uniquely upon mechanical valving by guard cells to regulate the movement of water vapour and CO_2_ between leaves and the atmosphere. The rapid and complex movements of guard cells make them favoured subjects for investigating signal transduction in plant cell membranes, while their critical global significance in regulating the entry of atmospheric carbon into terrestrial ecosystems adds an imperative to the goal of understanding how guard cell movements are controlled. Principal among guard cell movements is the tendency for stomata to respond rapidly to changes in CO_2_ concentrations inside the leaf, such that pores open with falling CO_2_ and close as CO_2_ concentrations rise [1]. Stomatal sensitivity to CO_2_ reflects a feedback between photosynthesis and stomatal aperture that allows efficient use of water [2], but also makes stomata sensitive to atmospheric concentrations of CO_2_. This sensitivity has major global ramifications while atmospheric concentrations of CO_2_ continue to soar [3]. 

Stomatal closure in response to rising CO_2_ has major ecological effects such as reduced canopy transpiration [4] and increasing water use efficiency during photosynthesis [5–7]. These patterns have been recorded in a great number of angiosperm species, but it has been known for some time that the stomata of conifer species do not dynamically close under elevated CO_2_ [7–9]. Considering the ecological implications of differential transpiration, photosynthesis and growth in response to rising CO_2_, the likelihood that the only two existing clades of tree species may have fundamentally different responses to elevated CO_2_ is of great significance [10]. Thus we seek to identify the mechanisms behind this evolutionary differentiation in the stomatal response to CO_2_ in vascular plants.

Two processes are considered the main drivers of stomatal responses to CO_2_; one is linked to photosynthesis [11–14] while the other involves the interaction of CO_2_ with guard cell membrane channels, via a Ca^2+^-dependent signalling pathway [15–19]. Evidence for photosynthesis-dependent stomatal responses to CO_2_ originated in studies comparing the responses of stomata from isolated epidermis not photosynthesising, with those from live leaves [11,12,20–22]. These studies consistently show diminished stomatal responses in isolated epidermis compared to live leaves [12]. The mechanism for photosynthesis-dependent stomatal responses to CO_2_ remains unknown, but a signal generated by photosynthesising mesophyll cells is believed to be transmitted to the guard cells in the apoplast [12]. In contrast to the photosynthesis-dependent stomatal responses to CO_2_, the activation of guard cell anion channels by increased CO_2_ has received significantly more attention facilitated by the comprehensive analysis of key guard cell signalling mutants [15–19,23,24]. These studies have presented compelling evidence that the guard cell signalling pathways of both intercellular Ca^2+^ and the phytohormone abscisic acid (ABA) converge on the closing response of stomata to elevated CO_2_, with the absence of either Ca^2+^ or ABA resulting stomata that are unable to close in response to an increase in CO_2_ [17,25]. Critical signalling proteins for this response of stomata to increased CO_2_ are the CALCIUM-DEPENDENT PROTEIN KINASEs (CDPKs) which phosphorylate guard cell anion channels causing membrane depolarisation and stomatal closure in the presence of elevated Ca^2+^, CO_2_ and ABA [19,26–28]. Current opinion favours a priming model for stomatal sensitivity to CO_2_ whereby stomatal responses to increased CO_2_ in angiosperms (but not conifers [29]) are enhanced by exposure of guard cells to either ABA or elevated Ca^2+^ [16,17,30].

These processes have been well characterised in angiosperms, but a number of studies have suggested that angiosperms may be the only group of land plants that close stomata in response to exposure to both short-term [7–9,29,31] and long-term [4,32] increases in CO_2_ concentrations above current atmospheric levels. Here we investigate the possibility that the Ca^2+^-dependent CO_2_ signalling pathway, which appears to be responsible for stomatal closure when CO_2_ rises above atmospheric concentrations [17], may have evolved after the divergence of the angiosperm lineage, more than 130 million years ago [33].

To evaluate the possibility that Ca^2+^-dependent signalling in guard cells only evolved in angiosperms we compared the CO_2_ responses of stomata in a diversity of vascular plants ranging from the early-diverging lycophyte clade to the more recent angiosperms. It was important to characterise stomatal CO_2_ responses under both light and dark conditions because stomata in angiosperms respond to CO_2_ via parallel pathways, one which interacts with the light activation of stomatal opening [12,14], and the other, light-independent pathway, associated with Ca^2+^-signalling [17,25]. Here we test two key hypotheses:

1. that the photosynthesis-dependent CO_2_ response pathway is present in all tracheophytes, and is only active at or below ambient atmospheric CO_2_ levels (approximately 400 µmol mol^-1^).
2. that only angiosperm stomata possess a Ca^2+^-signalling pathway in guard cells, operating in parallel with the photosynthesis-dependent pathway, which confers accelerated stomatal response times and sensitivity to CO_2_ concentrations both below and above ambient levels.

## Results and Discussion

### CO_2_ responses in the dark

**Figure 1:**
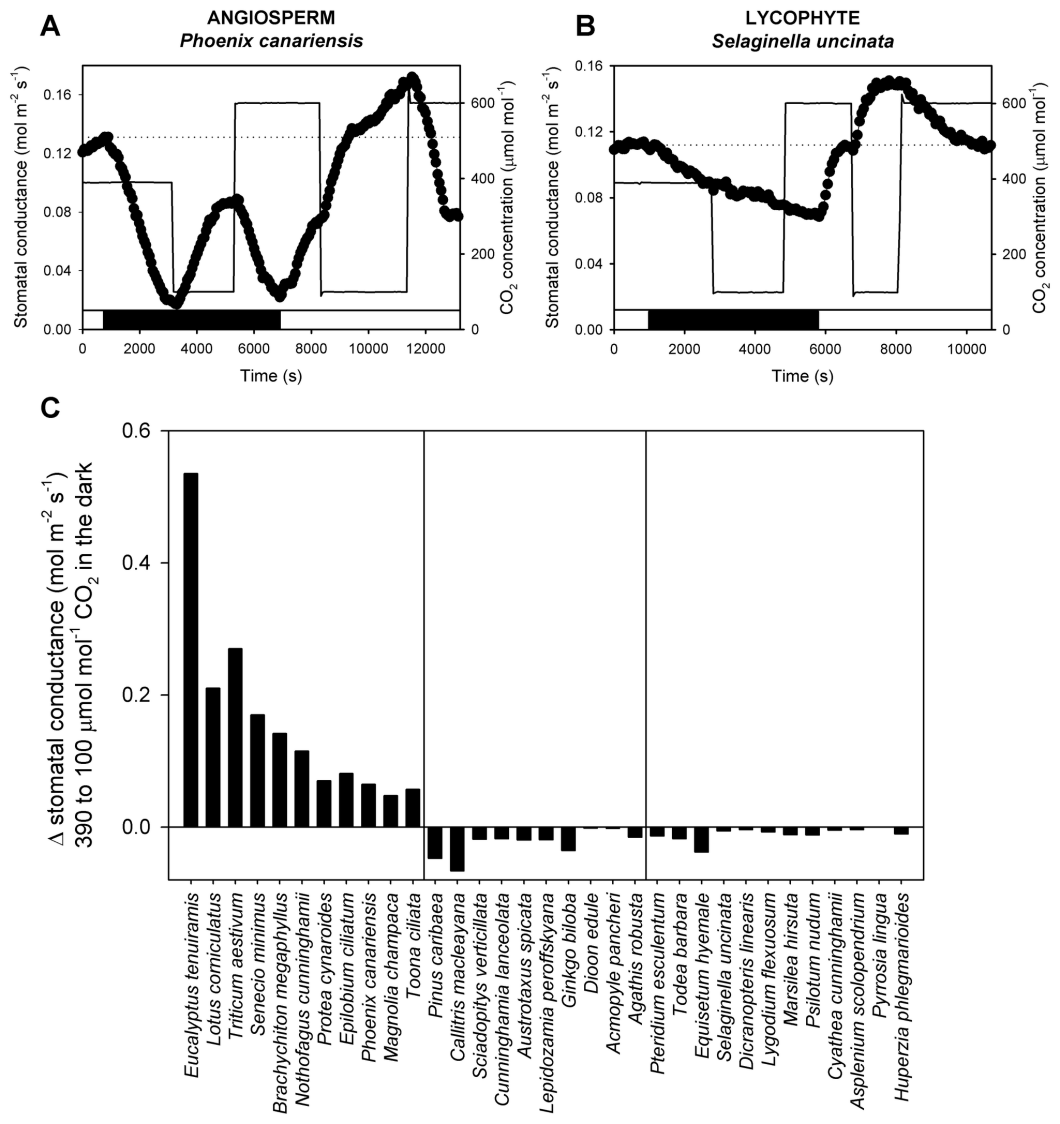
Only angiosperm stomata (i) respond to both low and high CO_2_ in the dark and (ii) when exposed to high CO_2_ in the light, close below stomatal conductances recorded at ambient CO_2_. The dynamic responses of stomatal conductance (black circles) are shown in leaves of a representative angiosperm (A) and lycophyte (B) subjected to transitions in ambient CO_2_ concentration (unbroken lines) in the light and dark (black horizontal bar), dotted horizontal lines indicate stomatal conductance at ambient atmospheric concentrations of CO_2_ in the light. Differences in stomatal opening between angiosperms and non-angiosperms exposed to low CO_2_ in the dark (C) were consistent within each diverse lineage of 11 angiosperm, 10 gymnosperm, 12 fern and lycophyte species, groups are separated by vertical lines.

**Figure 2:**
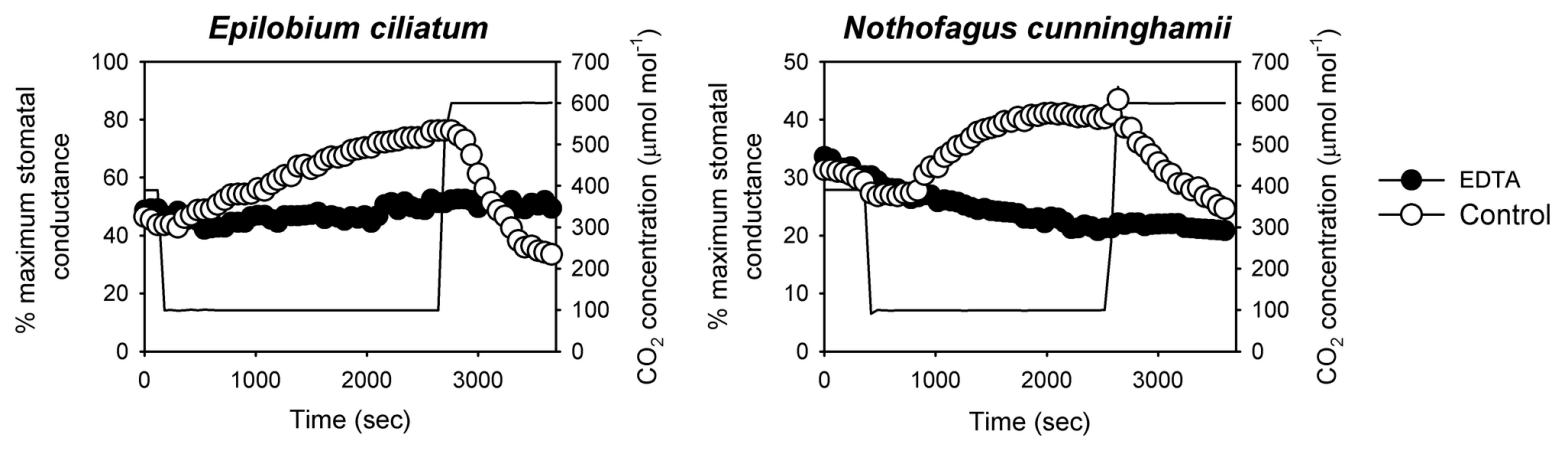
In the absence of calcium the stomata of angiosperms do not open in response to low CO_2_ in the dark. Dynamic changes in stomatal conductance of two angiosperms in response to a transition to low CO_2_ (continuous line) in the dark. Stomatal opening seen in excised leaves fed water (open circles) was eliminated by the presence of the mild calcium chelating agent ethylenediaminetetraacetic acid (EDTA, 10 mM) fed into the transpiration stream (closed circles). Values for maximum stomatal conductance in the light for each species are presented in Figure 3C.

Stomatal responses to CO_2_ in the dark are assumed to derive entirely from Ca^2+^-dependent signalling [17] because photosynthesis-dependent signalling is absent. We sampled 11 species of angiosperms from major families of eudicots and monocots (Table S1) and found that switching ambient CO_2_ concentration from atmospheric (400 µmol mol^-1^) to low (100 µmol mol^-1^) concentrations in the dark caused substantial stomatal opening in all species (Figure 1C). Declining CO_2_ concentration in the dark causes the deactivation of anion channels in guard cell plasma membranes leading to membrane polarisation and significant stomatal opening in model angiosperm species [34,35]. Subsequent transitions back to high CO_2_ (600 µmol mol^-1^) in the dark, led to the rapid closure of stomata in all angiosperm species examined (see example angiosperm species in Figure 1A), presumably due to the activation of anion channels in the guard cell plasma membrane [36] and a subsequent loss of guard cell turgor. We confirmed that stomatal responses to CO_2_ in the dark were associated with Ca^2+^-dependent signalling by showing that the introduction of a Ca^2+^ chelating agent (ethylenediaminetetraacetic acid , EDTA) into the transpiration stream of representative herbaceous and woody angiosperms eliminated the stomatal opening response to decreasing CO_2_ in the dark (Figure 2). 

**Figure 3:**
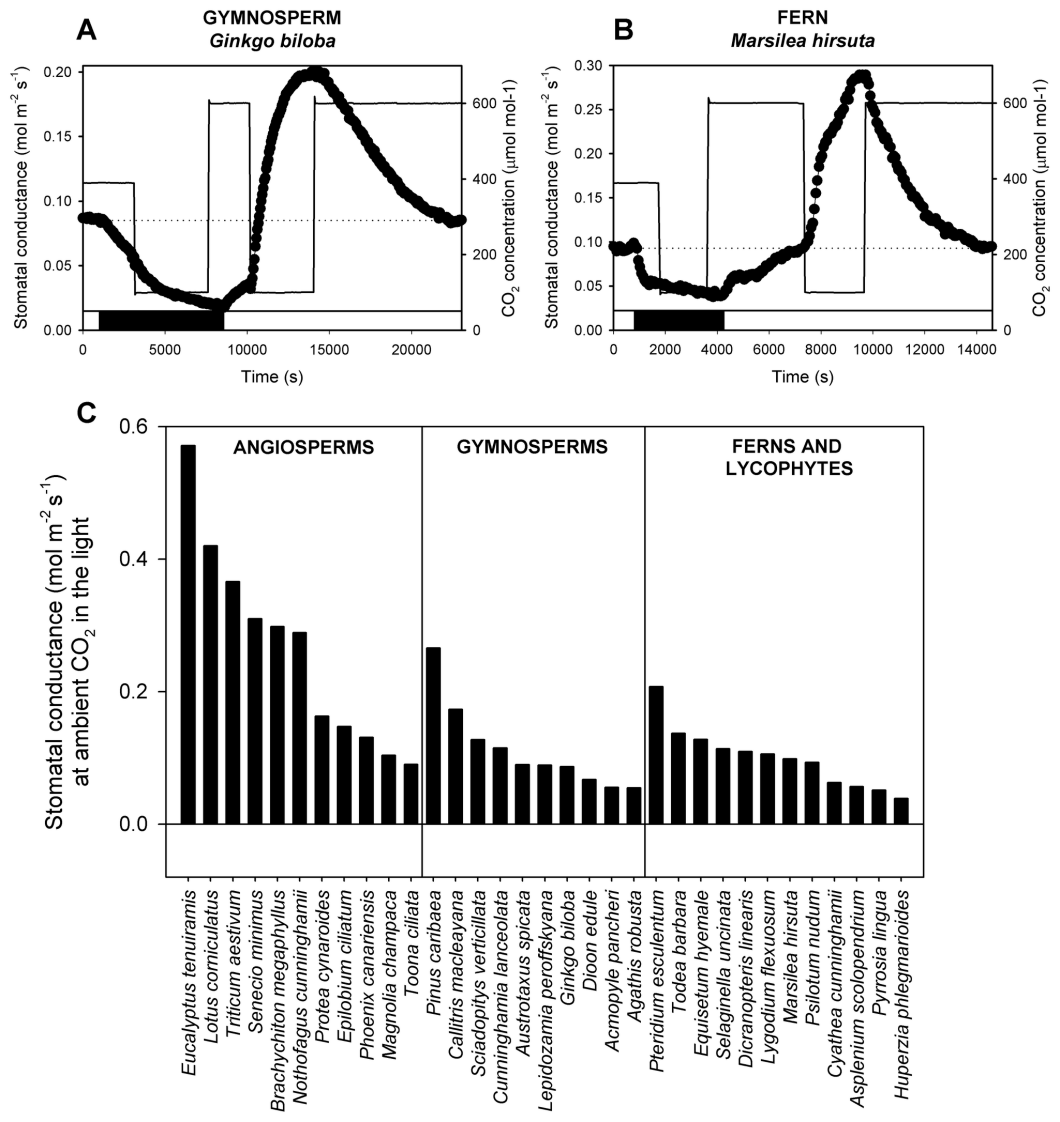
Similar to lycophytes (Figure 1B) the stomata of gymnosperms (A) and ferns (B) do not open in response to low CO_2_ in the dark and do not close at higher than ambient CO_2_ concentration in the light. (A and B) Dynamic changes in stomatal conductance (black dots) in response to changes in ambient CO_2_ concentration (thin solid line) in the dark and light (represented by black or white bars respectively at the bottom of the figure) in a representative gymnosperm *Ginkgo biloba* (A) and fern *Marsilea hirusta* (B). The dotted horizontal line represents stomatal conductance at ambient CO_2_ concentration in the light. (C) Maximum stomatal conductances in the light overlapped broadly among all species, divided into lineages by vertical lines.

In contrast to angiosperm species, we found no response to any change in CO_2_ concentration in the dark in our sample of 2 lycophytes, 10 fern and 10 gymnosperm species (Figures 1B, 3A and 3B). Stomata in the fern *Adiantum capillus-veneris* were previously shown to be non-responsive to CO_2_ in the dark [31] and our systematic sampling from 12 families of ferns and lycophytes, and 8 families of gymnosperms suggests that this response and thus Ca^2+^-signalling may be absent from the stomata of species from all non-angiosperm vascular plant lineages. 

### CO_2_ responses in the light

**Figure 4:**
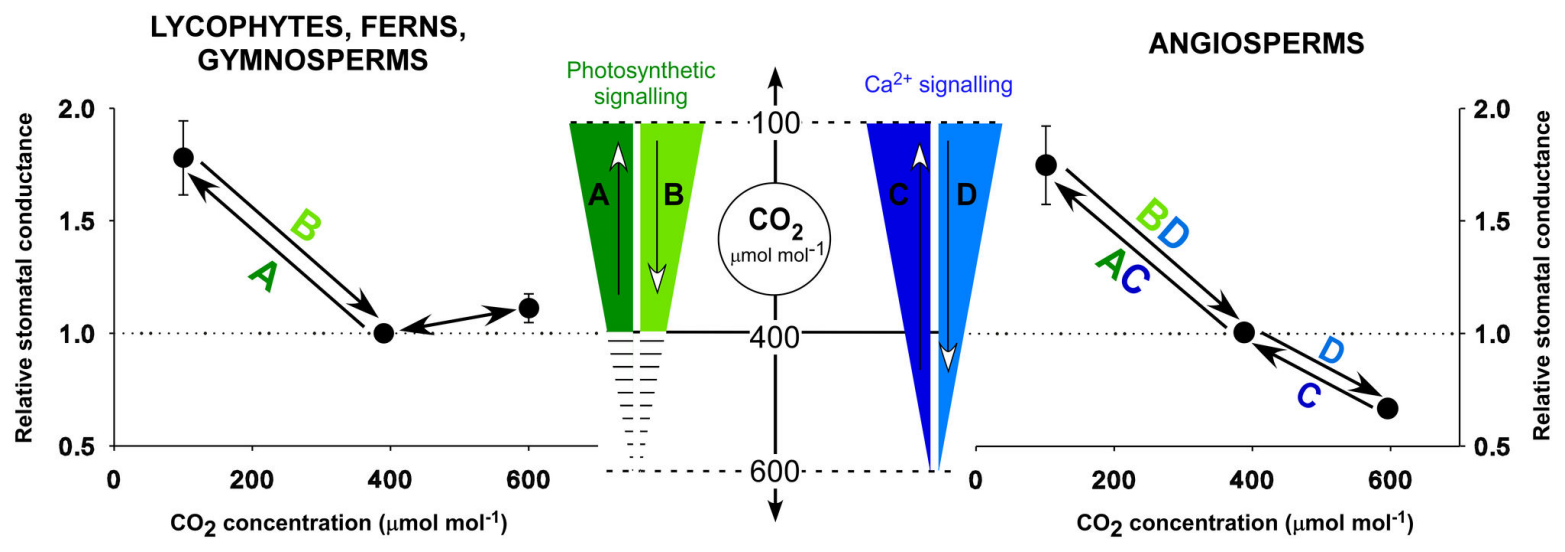
An evolutionary perspective on the interaction of the parallel pathways responsible for the CO_2_ sensitivity of angiosperm stomata. Stomatal conductances of all species were responsive to CO_2_ in the light, but the nature of these responses differed markedly in the angiosperms (n=11, ±SE) compared with the non-angiosperms (gymnosperms, ferns and lycophytes; n=22, ±SE). Values for stomatal conductance in the light at ambient CO_2_ concentration for each species are presented in Figure 3C. All species appear to possess the photosynthesis-dependent pathway (A and B, green icons), responsible for stomatal responses to changes in CO_2_ below current ambient atmospheric concentrations. In angiosperms only, stomatal responses to CO_2_ are driven by dual signals, coming from both photosynthetic signalling and the photosynthesis-independent Ca^2+^-signalling pathway (C and D, blue icons). The Ca^2+^-dependent pathway appears to be active across a range of CO_2_ concentrations above and below current atmospheric levels, leading to stomatal responses at CO_2_ concentrations above ambient levels (**C** and **D**), as well as conferring enhanced rates of stomatal response to CO_2_ (Figure 6C).

With the responses of stomata to CO_2_ in the dark suggesting an absence of Ca^2+^-signalling in non-angiosperm species, we subsequently investigated the possible impact of this absence on the responses of stomata to changes in CO_2_ in the light. Using a non-saturating light intensity, which allowed for opening responses of stomata at low CO_2_ concentrations to be realised, we found in all sampled species of lycophytes, ferns, gymnosperms and angiosperms, stomatal opening and closing responses to CO_2_ between transitions of low and ambient atmospheric CO_2_ concentrations in the light (Figure 4 and example gas exchange traces in Figures 1A, 1B, 3A and 3B). Despite a wide diversity in physiology, encompassing different stomatal conductances (Figure 3C) and photosynthetic rates at ambient CO_2_ in the light (Table S2), all sampled species had similar magnitudes of stomatal responses over the CO_2_ range of 100-400 µmol mol^-1^ (Figure 4). Angiosperm stomata, however, were distinct from the other species in possessing a stomatal response to transitions in CO_2_ above current atmospheric concentrations (Figure 4). As reported previously for lycophytes, ferns and conifers [7–9] and confirmed here, no significant decline in stomatal conductance was observed when ambient CO_2_ was increased from atmospheric concentration (400 µmol mol^-1^) to 600 µmol mol^-1^ (Figure 4 and example gas exchange traces in Figures 1A, 1B, 3A and 3B) in species representatives from any clade other than angiosperms. In angiosperms stomatal closure at elevated CO_2_ has been linked to Ca^2+^-dependent signalling [17], the asymmetry in the CO_2_ responsiveness of stomata in the light that we show for non-angiosperms, just like the absence of stomatal responses to CO_2_ in the dark, can be explained if stomatal specific Ca^2+^-dependent signalling evolved in a common ancestor of the modern angiosperms [17]. These two lines of evidence suggest that the universal sensitivity of stomata to sub-atmospheric CO_2_ transitions in the light derives from a photosynthesis-dependent signalling that is ancestral in vascular plants [31,37] while an origin of Ca^2+^-dependent stomatal signalling, only in angiosperms, explains their unique sensitivity to CO_2_ concentrations above current atmospheric levels (Figure 4).

### Ca^2+^- dependent signalling and ABA sensitivity

**Figure 5:**
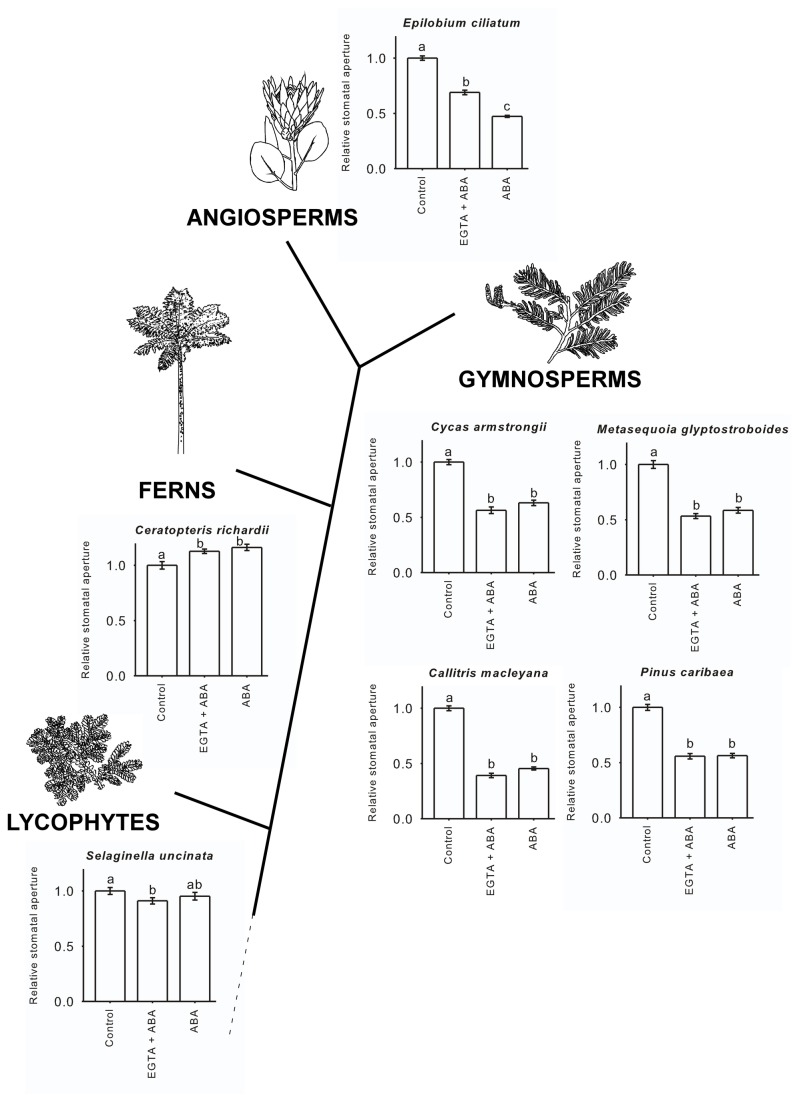
Increasing complexity in the evolution of stomatal responses to ABA, with the first response to ABA, in the gymnosperms, dominated by Ca^2+^-independent signalling, with functional Ca^2+^-dependent signalling evolving in angiosperms. Responses of stomatal apertures on excised epidermis to ABA (0.1 µM, in the angiosperm and gymnosperm species *Callitris macelyana* and *Cycas armstrongii*, 7.5 µM in the remaining gymnosperms, fern and lycophyte species) with and without the calcium specific chelator EGTA (10 mM). Three distinct patterns are evident. The fern and lycophytes group did not respond significantly to ABA; the gymnosperm representatives responded to ABA, but this response was not attenuated by the removal of Ca^2+^ by the addition of EGTA; while angiosperm stomata responded less to ABA when Ca^2+^ was removed by addition of EGTA [23,53,54]. Data are presented as means relative to open stomatal apertures in the control sample for n=50 live stomata from two leaves, viability was determined by fluorescence of the guard cells and all adjacent epidermal pavement cells following staining with fluorescein diacetate immediately prior to aperture measurements. All experiments were conducted double-blind, whereby the photographer as well as the measurer of stomatal apertures did not know either the species or treatment applied. Different letters denote significant difference (P<0.05).

Several recent studies suggest that the stomata of lycophytes and ferns are functionally non-responsive to the key phytohormone abscisic acid (ABA) [38,39]. Combined stomatal insensitivity to elevated CO_2_ and ABA in ferns and lycophytes is consistent with an absence of Ca^2+^-signalling in these clades (but see 40), considering that both responses converge upon the same Ca^2+^-dependent pathway to effect stomatal closure in angiosperms [26,27,41]. Unlike lycophytes and ferns, the stomata of gymnosperms are highly responsive to ABA [42], this is possible as the Ca^2+^ *-*dependent pathway is only one of two known signalling pathways for ABA activation of anion channels in the guard cells [23,26]. In angiosperms, the Ca^2+^ specific chelator ethylene glycol-bis(2-aminoethylether)-N,N,N′,N′-tetraacetic acid (EGTA) attenuates the ABA response of stomata by reducing the activity of the Ca^2+^-dependent pathway [23] while leaving the alternative pathway, signalling through the Ca^2+^-independent protein kinase, OPEN STOMATA1 (OST1) [43], unaffected (Figure 5). We found that the ABA response of representative gymnosperm stomata lacked the Ca^2+^-dependent sensitivity to ABA that characterises angiosperm stomata, providing a third line of evidence in support of the hypothesis that the Ca^2+^-dependent signalling pathway in gymnosperm guard cells is either absent or non-functional (Figure 5).

### Evolution of genes responsible for Ca^2+^-dependent signalling and transduction in stomata

**Figure 6:**
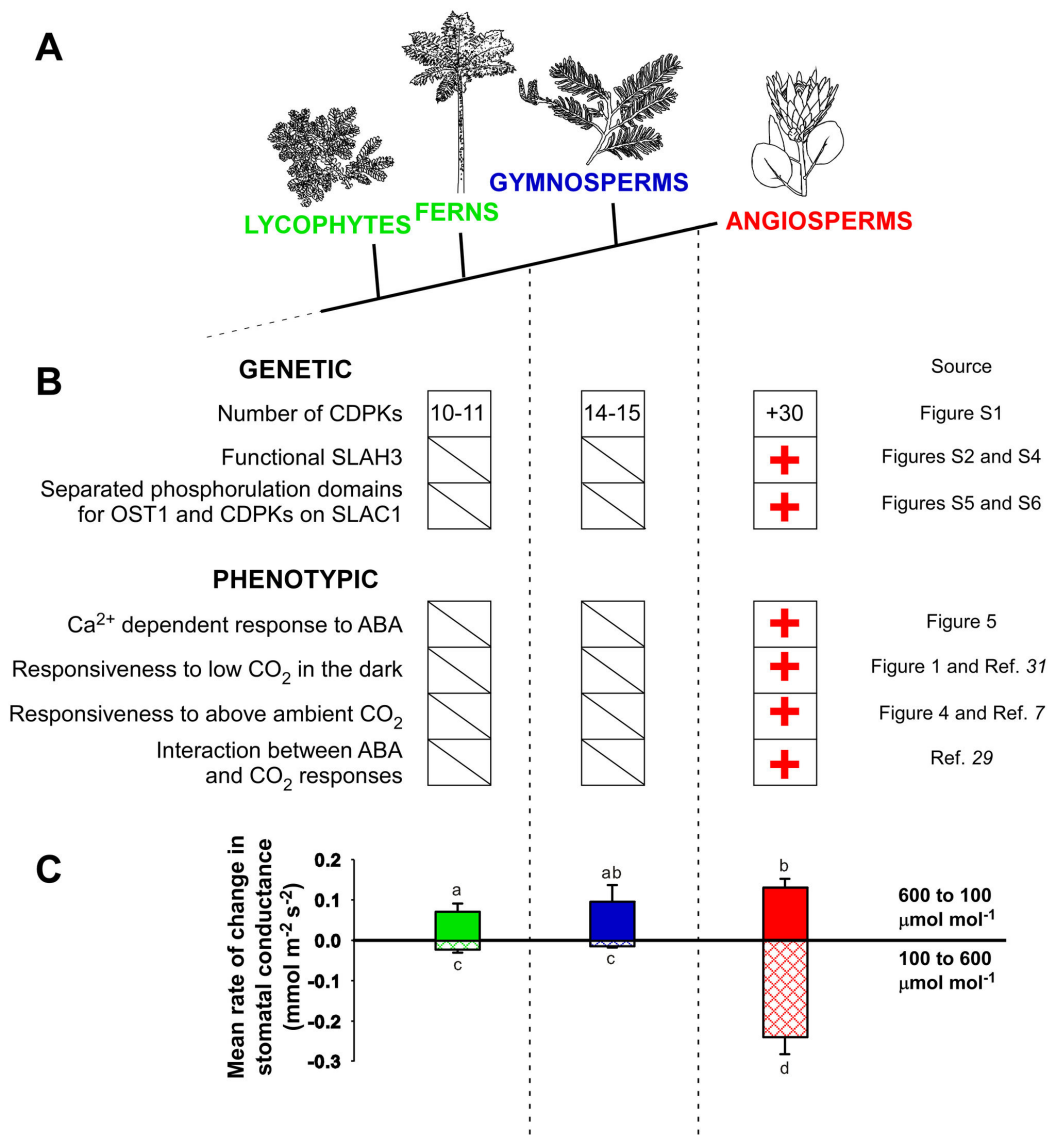
Summary of the key genetic and phenotypic characters supporting an origin of Ca^2+^-dependent stomatal signalling in the angiosperm clade. (A) Phylogenetic relationships between vascular plant groups, with the proposed evolution of Ca^2+^-dependent stomatal signalling within the angiosperm clade. (B) Results of searches (see Figures S1 and S4) for the genetic prerequisites for Ca^2+^-dependent stomatal signalling in the genomes of a representative gymnosperm (*Picea abies*) and lycophyte (*Selaginella moellendorffii*). As well as possessing many more CDPKs (Figure S1), angiosperms appear to have evolved several characters explicitly associated with Ca^2+^-dependent stomatal signalling. These innovations include a Ca^2+^-activated S-type anion channel (SLAH3) (Figures S2 and S4), and a dedicated CDPK phosphorylation site on the principal stomatal anion channel (SLAC1) (Figures S5 and S6). The resultant stomatal phenotype in angiosperms shows four key distinctions from other vascular plants in the stomatal response to CO_2_. (C) Faster dynamic stomatal closure in response to a step rise in ambient CO_2_ was observed in our angiosperm sample group (n= 11, ±SE) compared with combined lycophytes and ferns (n=12, ±SE) and gymnosperms (n=10, ±SE), this was unlike statistically similar rates of stomatal opening across groups following a step drop in ambient CO_2_, different letters denote significant difference (P<0.05). This suggests that Ca^2+^-dependent stomatal signalling provides angiosperms with an adaptive advantage by allowing faster dynamic stomatal closure in response to changing leaf internal CO_2_, thereby enabling more efficient use of water.

A central component to the function of Ca^2+^-signalling in angiosperm stomata are a diversity of CDPKs (29 in *Oryza* [44] and 34 in *Arabidopsis* [45]) and a recently identified Ca^2+^-activated S-type anion channel (SLAH3) [26]. We searched for sequences related to these critical genes in non-angiosperms by interrogating the genome sequences of the lycophyte *Selaginella moellendorffii* [46] and the gymnosperm *Picea abies* [47]. Unlike the diverse radiations seen in angiosperms we found only 14-15 CDPK genes in the gymnosperm and 10-11 in the lycophyte genome, none of which are closely related to the 7 stomatal-implicated *Arabidopsis* CDPKs (Figure S1). In addition we found that the protein SLAH3 in *Arabidopsis* [26] is not present in a functional form in either of the lycophyte or gymnosperm genomes (Figure S2). These data suggest that the evolution of Ca^2+^-dependent stomatal function in angiosperms has its origins in multiple duplications of Ca^2+^-signalling genes, as well as the evolution of a CDPK-dedicated stomatal anion channel (Figure 6). Further work is required for *in planta* analysis of possible stomatal specific CDPK and anion channel function in these non-angiosperms.

### Evolution in rates of stomatal response

If Ca^2+^-dependent guard cell signalling is unique to angiosperms, this raises the question, what advantage does this parallel pathway in CO_2_ response provide over the universal photosynthesis-dependent stomatal response? The most likely explanation is that the addition of the Ca^2+^-dependent pathway to guard cell signalling accelerates stomatal response time, specifically the rate of closure, thus enhancing the capacity of angiosperms to dynamically optimise diurnal water use [38]. Investigating this potential benefit we examined the speed of stomatal closure in response to a step increase in CO_2_ from 100 to 600 µmol mol^-1^. A very clear difference in closure kinetics was observed, with the stomata of species from the angiosperm group closing significantly faster than the stomata of the lycophyte, fern and gymnosperm species (Figure 6C). These differences between groups were specific to stomatal closing kinetics, and not due to any intrinsic sluggishness of lycophyte, fern or gymnosperm stomata, as illustrated by the fact that the mean rates of stomatal opening in gymnosperms were not significantly different to the angiosperm sample mean (Figure 6C). The specific contribution of Ca^2+^-dependent signalling to the speed of angiosperm responses has been shown in *Arabidopsis* mutants, where impaired Ca^2+^-signalling mutants show substantially reduced rates of stomatal closure at high CO_2_ [17], more akin to those of the lycophytes, ferns and gymnosperms measured here. Hence the superior gas exchange efficiency noted in angiosperms relative to other vascular plant groups [38], may be in part due to the evolution of the Ca^2+^- dependent pathway. 

Given that the Ca^2+^-dependent signalling pathway in guard cells causes stomatal closure at CO_2_ concentrations above about 400 µmol mol^-1^, it is not surprising that such a mechanism should be functionally absent in clades of vascular plants that evolved and radiated when atmospheric CO_2_ concentrations were likely to have been above 1000 µmol mol^-1^ [48]. On this basis it is reasonable to suggest that this mechanism proliferated, along with other adaptations in angiosperms suited to declining CO_2_ such as increasing leaf vein density [49] and decreasing stomatal size [50] as atmospheric CO_2_ concentrations fell during the angiosperm radiation late in the Cretaceous [48]. Assuming the benefits of Ca^2+^-dependent guard cell signalling are associated with increasing water use efficiency, a background of declining CO_2_ would increase selective pressure for this trait, as CO_2_ for photosynthesis became relatively more costly in terms of water loss [2]. There are however some unexpected implications to this line of reasoning, namely that the current era of rapidly rising CO_2_ may differentially depress the ability of angiosperms to realise higher rates of photosynthesis relative to their major woody competitors, the conifers, which apparently lack Ca^2+^-dependent stomatal signalling and hence do not close stomata as atmospheric CO_2_ rises. 

The idea that photosynthesis and growth in angiosperms may be attenuated relative to conifers in the current environment of rapidly rising atmospheric CO_2_ is supported by observations of gas exchange trends over time [51] and in free air CO_2_ enrichment studies [32]. There are reports for growth in conifers increasing significantly more than angiosperm species under increasing atmospheric CO_2_ levels [52]. We conclude that although rapid stomatal responses to increasing CO_2_ in angiosperms are likely to provide benefits in terms of an increased capacity to dynamically optimise water use during photosynthesis, the artefact that this increased responsiveness creates, whereby stomata are forced to close under rapidly increasing atmospheric CO_2_, creates a disadvantage for angiosperms relative to gymnosperms. This apparent cost of Ca^2+^-dependent stomatal signalling is unlikely to have been exposed to selection during the last 130 million years of angiosperm evolution [33], but under the current scenario where atmospheric CO_2_ levels will rise by >10% within a single generation of many tree species, it seems likely that angiosperms may suffer an unprecedented reduction in competitiveness due to their evolutionary innovation in stomatal function.

## Materials and Methods

### Species selected and growth conditions

The responses of stomata to instantaneous changes in ambient CO_2_ concentration (*C*_*a*_) as well as stomatal aperture experiments were observed in a wide diversity of species encompassing an evolutionary cross-section of vascular land plant lineages. To determine general patterns of stomatal behaviour within major clades of vascular plants, species were selected from key families of the four extant lineages of vascular plants; angiosperms, gymnosperms, ferns and lycophytes (Table S1). All species were grown as potted individuals housed in the glasshouses of the University of Tasmania under a 16-h photoperiod of natural light supplemented by sodium vapour lamps, ensuring a minimum 300 µmol quanta m^-2^ s^-1^ at the leaf surface. Temperatures in the glasshouse were maintained at 22°C during the day and 15°C at night. All plants were watered daily and fertilized with liquid nutrient weekly.

### Response of stomatal conductance to C_a_ in the dark and light

In 11 angiosperms, 10 gymnosperms, 10 ferns and 2 lycophytes (Table S1) an infrared gas analyser (Li6400; Li-Cor, Lincoln, NE, USA) was used to measure changes in stomatal conductance to water vapour (*g*_*s*_) in response to a series of transitions in ambient CO_2_ concentration in the light and dark. All other cuvette conditions remained constant; leaf temperature was maintained at 22°C, vapour pressure difference between 1.1 and 1.2 kPa and the chamber flow rate at 500 ml min^-1^. The *C*_*a*_ in the leaf cuvette was controlled for the duration of the experiment by a computer-controlled CO_2_ injection system (Li6400-01; Li-Cor, Lincoln, NE, USA). Leaf gas exchange and environmental parameters were automatically logged every minute. Leaves were enclosed in the cuvette and allowed to equilibrate at the current atmospheric *C*_*a*_ (400 µmol mol^-1^) and a light intensity of 300 µmol quanta m^-2^ s^-1^. This non-saturating light intensity ensured that maximum *g*_*s*_ was not reached when leaves were exposed to current atmospheric *C*_*a*_ ensuring an observation of stomatal opening at low *C*_*a*_ in the light. Following *g*_*s*_ stability (defined as less than a 5% change over 8 min) the light source in the cuvette was turned off, and after a period of approximately 20 min (or longer in ferns and lycophytes ensuring at least a 40% reduction in *g*_*s*_) in the dark, *C*_*a*_ was lowered to 100 µmol mol^-1^. To eliminate the possibility that low *g*_*s*_ in the lycophyte, fern and gymnosperm species may have prevented changes in *C*_*a*_ from being sensed inside the leaf, we ensured that dark transitions from ambient to low *C*_*a*_ were made at values of *g*_*s*_ sufficiently high to cause leaf internal CO_2_ concentrations to approach the target of 100 µmol mol^-1^ (Figure 1; Table S2). Regardless of the initial *g*_*s*_ we found that low *C*_*a*_ did not change the closing trajectory of stomata in the lycophyte, fern and gymnosperm species when lights were switched off (Figure 1). Following a period of at least 25 minutes at low *C*_*a*_ in the dark, which corresponded to the maximum time for *g*_*s*_ to increase and reach stability in the sample angiosperm species, *C*_*a*_ was increased to 600 µmol mol^-1^. This period of time was deemed sufficient to capture any possible delayed response of stomatal opening at low *C*_*a*_ in the dark in the lycophyte, fern and gymnosperm species, with the stomata of the lycophyte *Selaginella uncinata* showing no sign of stomatal opening after an extended period of an hour exposed to low *C*_*a*_ in the dark (Figure S3). Leaves were maintained at a 600 µmol mol^-1^ *C*_*a*_ in the dark until *g*_*s*_ had reached stability, following which the light intensity was again increased to 300 µmol quanta m^-2^ s^-1^. In the lycophyte, fern and gymnosperm species with the absence of any change in slope in the progressive decline of *g*_*s*_ following the exposure of leaves to darkness, the light intensity was increased to 300 µmol quanta m^-2^ s^-1^ after at least 10 minutes. Leaves were exposed to 600 µmol mol^-1^ *C*_*a*_ in the light until *g*_*s*_ had reached stability following opening. Following opening in the light *C*_*a*_ was then lowered to 100 µmol mol^-1^ and finally increased to 600 µmol mol^-1^ allowing *g*_*s*_ to reach stability after each transition. All gas exchange parameters were corrected to account for the leaf area in the cuvette.

### Response of angiosperm stomata to low C_a_ in the dark in the presence of a calcium chelator

To investigate the possible role of guard cell Ca^2+^-signalling in the opening response of angiosperm stomata to low *C*_*a*_ in the dark, leaf gas exchange was measured in the angiosperm tree species *Nothofagus cunninghamii* and herb *Epilobium ciliatum* following a series of transitions in light intensity and *C*_*a*_ in the presence of the mild calcium chelating agent ethylenediaminetetraacetic acid (EDTA) fed into the transpiration stream. Stems of both species were excised under resin filtered, deionised water and leaves were enclosed in the cuvette of a gas analyser (as described above) at ambient *C*_*a*_ and a light intensity of 300 µmol quanta m^-2^ s^-1^ (all other leaf environmental conditions were maintained as described above and gas exchange and cuvette environmental data automatically logged every minute). Following stability in *g*_*s*_, lights in the cuvette were turned off, and after 30 min *C*_*a*_ was lowered to 100 µmol mol^-1^ and maintained for a further 30 min following which *C*_*a*_ was increased to 600 µmol mol^-1^. After 20 min at high *C*_*a*_ in the dark an aliquot of EDTA was added to the deionised water ensuring a concentration of 10 mM entering the transpiration stream, concurrently *C*_*a*_ was lowered to ambient levels and light intensity increased to 300 µmol quanta m^-2^ s^-1^. Following 20 minutes of EDTA feeding, at the point when stomata began to slowly close due to the removal of calcium, the cuvette light was turned off. After a 50% reduction in *g*_*s*_, *C*_*a*_ was again lowered to 100 µmol mol^-1^ and maintained for a further 30 min after which *C*_*a*_ was increased to 600 µmol mol^-1^. Following this transition the light in the chamber was again increased to 300 µmol quanta m^-2^ s^-1^ to ensure stomata were able to open following the addition of EDTA into the transpiration stream.

### Stomatal aperture responses to ABA

A sub-sample of species was chosen for detailed measurements of stomatal aperture responses by light microscopy. Seven species were chosen to represent the four extant vascular plant lineages, including one angiosperm, four gymnosperms, a fern and a lycophyte (Table S1). Only a single angiosperm was sampled because stomatal aperture responses in this clade are well known, in contrast to the other groups [23,53,54]. To analyse the responses of live guard cells in intact epidermes, the most recently fully expanded leaf was used for each species, with leaf epidermes carefully removed with a razor blade and fine forceps under resin filtered, deionised water. Wax stomatal plugs were removed from the epidermes of *Pinus caribaea* with the non-toxic, putty Blu-Tack (Bostick, Australia) according to the methods of Feild et al. [55]. Epidermes were incubated for 1 h in a petri dish containing control buffer (50 mM KCl, 10 mM MES, pH 6.15, rendered nominally CO_2_ free following 1 h of bubbling with N_2_ gas) in the light (200 µmol quanta m^-2^ s^-1^). At least six epidermal strips were prepared per species, and at least two were transferred to each of a control buffer that contained added abscisic acid (ABA) and calcium (CaCl_2_, 0.1 mM), or a control buffer (without added CaCl_2_) that contained the same concentration of ABA and the calcium specific chelator ethylene glycol-bis(2-aminoethylether)-N,N,N′,N′-tetraacetic acid (EGTA, 10 mM). The level of ABA used was determined by prior dose-response experiments as the level that resulted in approximately 50% stomatal closure in the seed plant species; with 0.1 µM used in the angiosperm species and two of the gymnosperms *Callitris macleyana* and *Cycas armstrongii*, and 7.25 µM used for the two gymnosperm species *P*. *caribaea* and *Metasequoia glyptostroboides*. No stomatal response to ABA could be determined in the stomata of fern and lycophytes species, so a concentration of 7.25 µM ABA was used. Epidermes were also continually maintained in the control buffer. Once transferred, epidermes were further incubated in petri dishes containing the treatment or control buffers in the light for 2 h. Immediately (~2 min) prior to stomatal aperture measurements fluorescein diacetate dissolved in acetone was added to the treatment buffer (ensuring a concentration of 60 µM in the buffer solution) to enable the visualisation of live stomata. Epidermes were then transferred to a microscope slide in the treatment buffer and live guard cells only, determined by the vivid fluorescence of both guard cells and all adjacent epidermal pavement cells, were photographed under white light at a magnification of x40 (Axiocam, Carl Zeiss, Oberkochen, Germany). A double-blind measurement protocol, whereby the photographer and the measurer of stomatal apertures had no prior knowledge of either the species or treatment was applied and stomatal apertures determined using the program ImageJ (developed at the United States National Institutes of Health, http://rsbweb.nih.gov/ij/1). 

### Evolution of the CDPK and SLAC1/SLAH gene families

To investigate the evolution of both the CDPK and SLAC1/SLAH gene families in vascular land plants we performed Basic Local Alignment Search Tool (BLAST) searches for all of the known *Arabidopsis thaliana* CDPK, SLAC1 and SLAH genes in the sequenced genomes of the lycophyte *Selaginella* moellendorffii [46] at Phytozome (www.phytozome.net) and the recently published genome of the gymnosperm *Picea abies* [47] *at* Congenie (http://congenie.org). To confirm that all of the CDPK gene models were identified in these two species and to assess the relationships between these identified CDPKs and the CDPKs that have been shown to have stomatal function in angiosperms [26–28,30,56,57], a phylogram was created using distance and parsimony-based methods in PAUP (version 4.0b10, http://paup.csit.fsu.edu/) from an alignment performed with the programme ClustalX [58] using the results of BLAST searches that included not only CDPKs but also proteins from both species that included closely related protein kinases (Figure S1, Table S3). For the SLAC1/SLAH gene family, all identified proteins from BLAST searches of the *Picea* genome were used in addition to a majority of SLAC1/SLAH proteins already identified from angiosperm, *Selaginella* and moss (*Physcomitrella patens*) sequenced genomes [59] to construct a phylogram, as described above (Figure S4, Table S4). In *A. thaliana* the activation of the S-type anion channels SLAC1 and SLAH3 occurs via phosphorylation by either of the proteins OPEN STOMATA1 (OST1) or a number of CDPKs [26–28]. Many of these phosphorylation domains are known [26,27,60] and conserved across angiosperms [59]. We mapped the phosphorylation domain for CDPK21 on SLAH3 described by Geiger et al. [26]; the phosphorylation domain for CDPK6 on SLAC1 identified by Brand et al. [60]; and the phosphorylation domains for OST1 on SLAC1 identified by Geiger et al. [43] with all positions refined by Dreyer et al. [59], who also identified the transmembrane domains for SLAC1 as well as conserved phosphorylation sites on SLAC1 in angiosperms. We used protein sequence alignments to determine the presence or absence of these key phosphorylating domains in the candidate SLAC1 (Figure S5) and SLAH3 (Figure S2) proteins of *Picea* and *Selaginella* to provide information on the possible functionality and evolution of these important anion channel-protein interactions observed in angiosperms. We did not include the recently identified interactions between SLAH3 and CDPK21 in *Arabidopsis* that has, in addition to the above described phosphorylation domains, been shown to be regulated by ABA through plasma membrane nanodomains [61].

## Supporting Information

Figure S1 **Evolution of the CDPK gene family in vascular plants.** Phylogenetic relationships based on amino-acid sequences of all *Arabidopsis thaliana* (At, red), *Picea abies* (Pa, blue) and *Selaginella moellendorffii* (Sm, green) calcium dependent protein kinases (CDPKs) as well as a selection of closely related protein kinases to the CDPKs (all black) including the CDPK-related kinases (CRKs) and phosphoenolpyruvate carboxylase kinases (PPCKs) (of which two are from the angiosperm species *Oryza sativa* (Os) and *Mesembryanthemum crystallinum* (Mc)) with the tree rooted to the phosphoenolpyruvate carboxylase kinase-related kinases (PEPRKs) and SNF-1 related kinases (SNRKs). Angiosperms are characterised by an abundance of CDPKs compared with lycophytes and conifers [44,45]. Of the AtCDPKs that are expressed in guard cells and have been shown to have specific anion channel function (thick red branches with shadowed names) [26,27,28,30,56,57] none are closely related to any *Picea* or *Selaginella* CDPK, and often occur in distinctive *Arabidopsis* only clades of CDPKs. Bootstrap values from 1000 trees are shown above or next to each branch. Sequence annotation details can be found in Table S3.
(TIF)

Figure S2 **Likely non-functional anion channel SLAH3 in the gymnosperm *Picea*. Alignment of the *Arabidopsis thaliana* (At) SLAC1-homologue 3 (SLAH3) protein sequence with the only SLAH3-like protein identified by BLAST searches of the recently published genome of *Picea abies* (Pa) (Figure S4).** The protein in *Picea abies* is predominantly expressed in buds (http://congenie.org). Below the alignment is shown the putative *Arabidopsis* phosphorylation domain for AtCDPK21 [26] in blue. Note the absence of not only this phosphorylation domain but also a large portion of the AtSLAH3 protein sequence in the *Picea* SLAH3-like sequence. Shading in the sequence alignment indicating the degree of amino acid conservation (black = 100%, grey = 50%). Recently the interaction between SLAH3 and CDPK21 in *Arabidopsis* has additionally been shown to be regulated by ABA through plasma membrane nanodomains [61].
(PDF)

Figure S3 **The stomata of lycophytes do not respond to low CO_2_ in the dark over an extended period of time.** The stomatal response (black circles) of the lycophyte *Selaginella uncinata* to exposure to low CO_2_ (black line) over an extended period in the darkness (indicated by the black horizontal bar).
(TIF)

Figure S4 **Evolution of the SLAC1 and SLAH gene family.** Phylogenetic relationships based on the amino-acid sequences of all slow anion channel 1 (SLAC1) and SLAC1-homologue (SLAH) of *Arabidopsis thaliana* (At), *Picea abies* (Pa), *Selaginella moellendorffii* (Sm), Physcomitrella patens (Pp) and other sequenced angiosperm species numbered (see Table S4 for a key to the sequence details). The tree is rooted to the most closely related proteins from algae [59], colours represent the respective functional groups [59].
(TIF)

Figure S5 **Limited phosphorylation domains for OST1 and CDPKs on the anion channel SLAC1 of the gymnosperm *Picea***. Alignment of the *Arabidopsis thaliana* (At) SLAC1 protein sequence and most similar sequences of SLAC1-like proteins identified by BLAST searches of the recently published genome of *Picea abies* (Pa) (Figure S4). The two SLAC1-like proteins identified in *Picea* are predominantly expressed in leaves (http://congenie.org). Below the alignment is shown the *Arabidopsis* phosphorylation domain for AtCDPK6 [60] in blue. The putative phosphorylation domains for AtOST1 previously identified [43] are also shown, with positions refined to the conserved domains documented in red. Yellow letters indicate conserved phosphorylation sites in angiosperms [59], red shading indicated phosphorylation sites that are not conserved in the *Picea* proteins, while the bold phosphorylation domain for AtOST1 denotes the predominant phosphorylating domain in *Arabidopsis* [43]. Note the lack of conservation of a number of key N-terminus AtOST1 phosphorylating domains in the two identified SLAC1-like proteins of *Picea*, particularly in PaMA_10428033p0010 which is the only SLAC1-like *Picea* protein to share a known CDPK phosphorylating domain. This suggests strong competition for this phosphorylating domain between PaOST1 and any possible stomatal associated PaCDPKs. Transmembrane motifs for SLAC1 are shown in green [59]. Shading in the sequence alignment indicating the degree of amino acid conservation (black = 100%, grey = 50%).
(PDF)

Figure S6 **Lack of all key N-terminus OST1 phosphorylating domains found in angiosperms on the putative *Selaginella* SLAC1 proteins.** Alignment of the *Arabidopsis thaliana* (At) SLAC1 protein sequence and the only four SLAC1-like proteins identified by BLAST searches of the genome of *Selaginella moellendorffii* (Sm) (Figure S4). Below the alignment is shown the *Arabidopsis* phosphorylation domain for AtCDPK6 in blue, while the putative phosphorylation domains for AtOST1 identified by Geiger et al. [43] (with positions refined to the conserved domains [59]) are shown in red. Yellow letters shaded in red indicate conserved phosphorylation sites in angiosperms [59], while the underlined phosphorylation domain for AtOST1 denotes the predominant phosphorylating domain in *Arabidopsis* [43]. Note the lack of conservation of all key N-terminus AtOST1 phosphorylating domains in all four identified SLAC1-like proteins of *Selaginella*. Transmembrane motifs for SLAC1 are shown in green [59]. Shading in the sequence alignment indicates the degree of amino acid conservation (black = 100%, dark grey = 80%, light grey = 60%).
(PDF)

Table S1 **Experimental species including family and a brief description of the native habitat and ecology.**
(DOCX)

Table S2 **Photosynthetic rates (µmol m^-2^ s^-1^) and internal leaf CO_2_ concentration (*C_i_*) at current ambient atmospheric CO_2_ concentration (400 µmol mol^-1^) and the ratio of *C*_*i*_ to *C*_*a*_ at low CO_2_ (100 µmol mol^-1^) in the dark for each experimental species.**
(DOCX)

Table S3 **Accession numbers and gene models of CDPKs and related protein kinase sequences in the conifer *Picea abies* (Pa) (including details on expressed tissue) lycophyte *Selaginella moellendorffii* (Sm) and angiosperms *Arabidopsis thaliana* (At), *Oryza sativa* (Os) and *Mesembryanthemum crystallinum* (Mc) used in the alignment and subsequent phylogram in Figure S1. Predicted amino acid sequence gene models and information on expressed tissue was identified in BLAST searches of the P. *abies* (http://congenie.org/) and *S. moellendorffii* (http://www.phytozome.net) genomes.** Angiosperm Genbank accession numbers are given for each of the protein sequences used in the alignment and phylogenetic neighbour-joining tree.
(DOCX)

Table S4 **Sequence information including species, gene model and source for the numbered protein sequences used to construct the phylogenetic neighbour-joining tree shown in Figure S4.**
(DOCX)
