# Supplementary figures and images for: Unique Responsiveness of Angiosperm Stomata to Elevated CO2 Explained by Calcium Signalling
Source: PLoS One. 2013 Nov 20;8(11):e82057. doi: 10.1371/journal.pone.0082057 (PMC3835710; doi:10.1371/journal.pone.0082057)

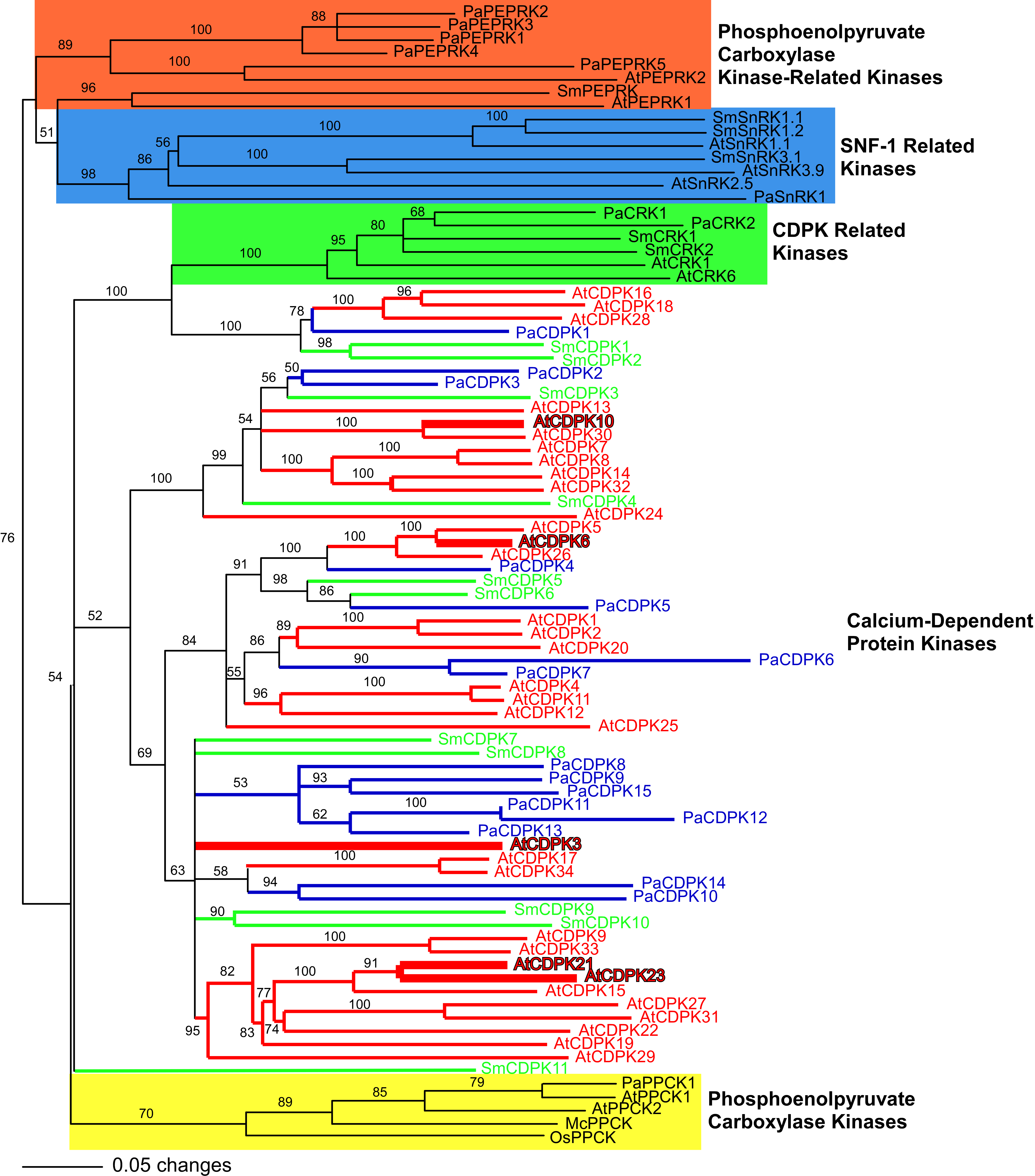

Supplement: Figure S1 — Evolution of the CDPK gene family in vascular plants. Phylogenetic relationships based on amino-acid sequences of all Arabidopsis thaliana (At, red), Picea abies (Pa, blue) and Selaginella moellendorffii (Sm, green) calcium dependent protein kinases (CDPKs) as well as a selection of closely related protein kinases to the CDPKs (all black) including the CDPK-related kinases (CRKs) and phosphoenolpyruvate carboxylase kinases (PPCKs) (of which two are from the angiosperm species Oryza sativa (Os) and Mesembryanthemum crystallinum (Mc)) with the tree rooted to the phosphoenolpyruvate carboxylase kinase-related kinases (PEPRKs) and SNF-1 related kinases (SNRKs). Angiosperms are characterised by an abundance of CDPKs compared with lycophytes and conifers [44,45]. Of the AtCDPKs that are expressed in guard cells and have been shown to have specific anion channel function (thick red branches with shadowed names) [26,27,28,30,56,57] none are closely related to any Picea or Selaginella CDPK, and often occur in distinctive Arabidopsis only clades of CDPKs. Bootstrap values from 1000 trees are shown above or next to each branch. Sequence annotation details can be found in Table S3. (TIF) [file pone.0082057.s001.tif]

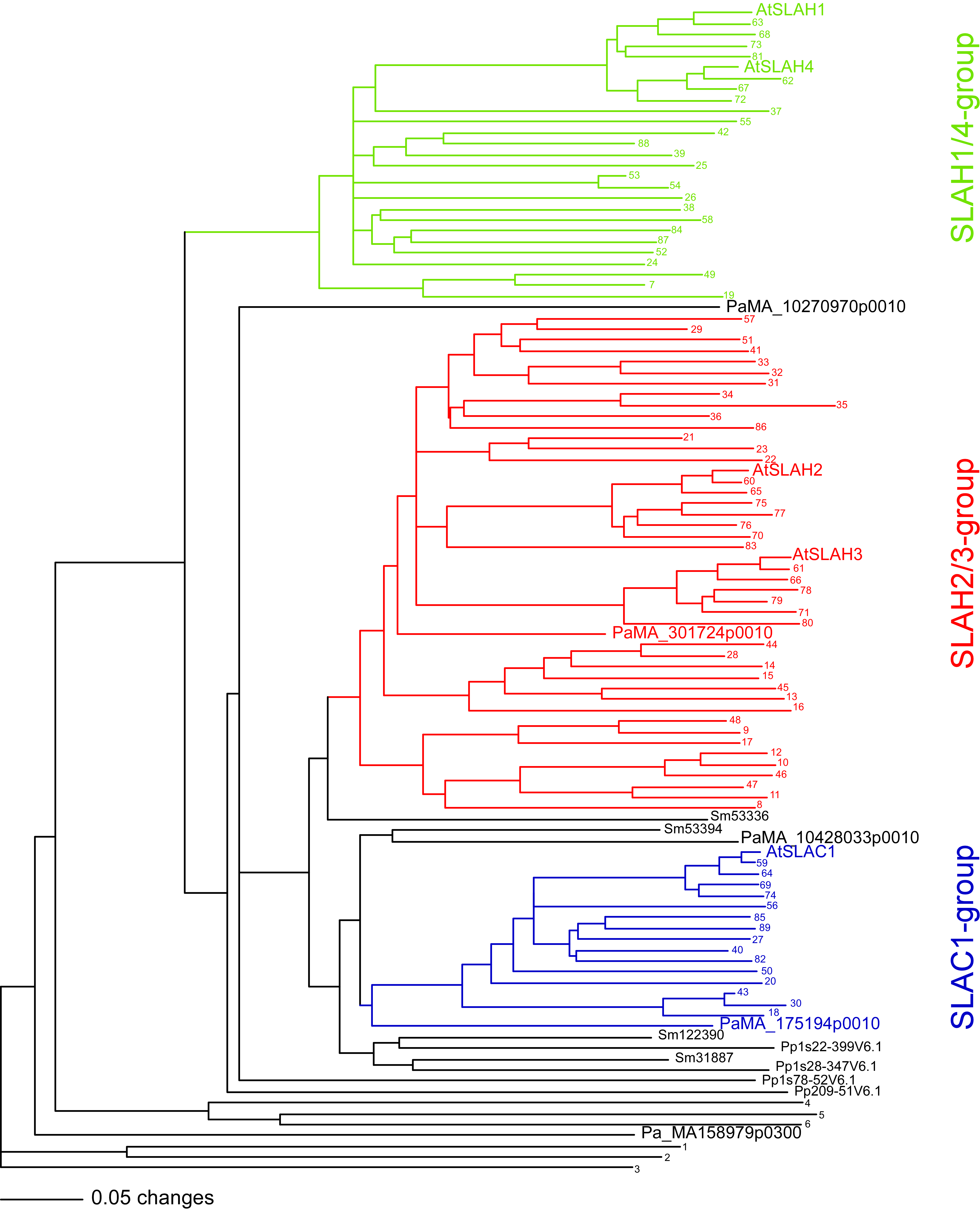

Supplement: Figure S4 — Evolution of the SLAC1 and SLAH gene family. Phylogenetic relationships based on the amino-acid sequences of all slow anion channel 1 (SLAC1) and SLAC1-homologue (SLAH) of Arabidopsis thaliana (At), Picea abies (Pa), Selaginella moellendorffii (Sm), Physcomitrella patens (Pp) and other sequenced angiosperm species numbered (see Table S4 for a key to the sequence details). The tree is rooted to the most closely related proteins from algae [59], colours represent the respective functional groups [59]. (TIF) [file pone.0082057.s004.tif]
