# Supplementary material for: Unique Responsiveness of Angiosperm Stomata to Elevated CO2 Explained by Calcium Signalling
Source: PLoS One. 2013 Nov 20;8(11):e82057. doi: 10.1371/journal.pone.0082057 (PMC3835710; doi:10.1371/journal.pone.0082057)
Supplement: Figure S2 — Likely non-functional anion channel SLAH3 in the gymnosperm Picea. Alignment of the Arabidopsis thaliana (At) SLAC1-homologue 3 (SLAH3) protein sequence with the only SLAH3-like protein identified by BLAST searches of the recently published genome of Picea abies (Pa) (Figure S4). The protein in Picea abies is predominantly expressed in buds (http://congenie.org). Below the alignment is shown the putative Arabidopsis phosphorylation domain for AtCDPK21 [26] in blue. Note the absence of not only this phosphorylation domain but also a large portion of the AtSLAH3 protein sequence in the Picea SLAH3-like sequence. Shading in the sequence alignment indicating the degree of amino acid conservation (black = 100%, grey = 50%). Recently the interaction between SLAH3 and CDPK21 in Arabidopsis has additionally been shown to be regulated by ABA through plasma membrane nanodomains [61]. (PDF) [file pone.0082057.s002.pdf]

```
                           *        20         *        40         *        60
PaMA_30172p0010 : ------------------------------------------------------------ :   -
AtSLAH3         : MEEKPNYVIQVEEELPTLLRKATTEEMVGFDNYKENGHPFPHSISRFHPSHASTTTLNGQ :  60

                           *        80         *       100         *       120
PaMA_30172p0010 : ------------------------------------------------------------ :   -
AtSLAH3         : ETSRSIDTMEAHHHNYNETTPWTHQRKPSISMPTSPNVLMISDPTTSLSSENHKNSGSTG : 120

                           *       140         *       160         *       180
PaMA_30172p0010 : ------------------------------------------------------------ :   -
AtSLAH3         : KSVKFLSQPMTKVSSLYIESGNGDDDRRQSHDNHHHHLHRQHQSGHHQNQNQAANKLKDN : 180
                                                                     ↑.........

                           *       200         *       220         *       240
PaMA_30172p0010 : ------------------------------------------------------------ :   -
AtSLAH3         : RYNSFKTWSGKLERQFTRKPASVEPEAPNRNNQNLNTNEAMPVDRYYDALEGPELETLRP : 240
                  .CDPK23......................↑

                           *       260         *       280         *       300
PaMA_30172p0010 : ------------------------------------------------------------ :   -
AtSLAH3         : QEEIVLPNDKKWPFLLRYPISTFGMCLGVSSQAIMWKTLATAEPTKFLHVPLWINQGLWF : 300

                           *       320         *       340         *       360
PaMA_30172p0010 : ------------------------------------------------------------ :   -
AtSLAH3         : ISVALILTIATIYLLKIILFFEAVRREYYHPIRINFFFAPFISLLFLALGVPPSIITDLP : 360

                           *       380         *       400         *       420
PaMA_30172p0010 : ----------------------MSGGSRRLSKVANPSNYLAIVGNFVGALLGATTGWNEG :  38
AtSLAH3         : HFLWYLLMFPFICLELKIYGQWMSGGQRRLSRVANPTNHLSVVGNFVGALLGASMGLREG : 420

                           *       440         *       460         *       480
PaMA_30172p0010 : AIFFFAVGLAHYLVLFVTLYQRLPTNEALPKELHPVFFLFVAAPSVASVAWEKIQGDFGY :  98
AtSLAH3         : PIFFYAVGMAHYLVLFVTLYQRLPTNETLPKDLHPVFFLFVAAPSVASMAWAKVTGSFDY : 480

                           *       500         *       520         *       540
PaMA_30172p0010 : VSKIAYFISLFLYTSLVVRLNFFRGFRFSITWWAYTFPMTGAAVATIKYSNKIRHPITQS : 158
AtSLAH3         : GSKVCYFIAIFLYFSLAVRINFFRGIKFSLSWWAYTFPMTGAAIATIRYATVVKSTMTQI : 540

                           *       560         *       580         *       600
PaMA_30172p0010 : LAVTLSIISSLTVFSLLLTTILHAFVWGTLFPNDISIAITHKKHKSKKNKK--------- : 209
AtSLAH3         : MCVVLCAIATLVVFALLVTTIIHAFVLRDLFPNDLAIAISNRPRPKQNSQHRWLDQLRNV : 600

                           *       620         *
PaMA_30172p0010 : SAEGKK---------------------DTLLSELSL : 224
AtSLAH3         : SSENIENYLKFTDSDSSQSNDVEACNGKTQESDSS- : 635
```
