# Supplementary material for: Unique Responsiveness of Angiosperm Stomata to Elevated CO2 Explained by Calcium Signalling
Source: PLoS One. 2013 Nov 20;8(11):e82057. doi: 10.1371/journal.pone.0082057 (PMC3835710; doi:10.1371/journal.pone.0082057)
Supplement: Figure S5 — Limited phosphorylation domains for OST1 and CDPKs on the anion channel SLAC1 of the gymnosperm Picea. Alignment of the Arabidopsis thaliana (At) SLAC1 protein sequence and most similar sequences of SLAC1-like proteins identified by BLAST searches of the recently published genome of Picea abies (Pa) (Figure S4). The two SLAC1-like proteins identified in Picea are predominantly expressed in leaves (http://congenie.org). Below the alignment is shown the Arabidopsis phosphorylation domain for AtCDPK6 [60] in blue. The putative phosphorylation domains for AtOST1 previously identified [43] are also shown, with positions refined to the conserved domains documented in red. Yellow letters indicate conserved phosphorylation sites in angiosperms [59], red shading indicated phosphorylation sites that are not conserved in the Picea proteins, while the bold phosphorylation domain for AtOST1 denotes the predominant phosphorylating domain in Arabidopsis [43]. Note the lack of conservation of a number of key N-terminus AtOST1 phosphorylating domains in the two identified SLAC1-like proteins of Picea, particularly in PaMA_10428033p0010 which is the only SLAC1-like Picea protein to share a known CDPK phosphorylating domain. This suggests strong competition for this phosphorylating domain between PaOST1 and any possible stomatal associated PaCDPKs. Transmembrane motifs for SLAC1 are shown in green [59]. Shading in the sequence alignment indicating the degree of amino acid conservation (black = 100%, grey = 50%). (PDF) [file pone.0082057.s005.pdf]

```
                              *        20         *        40         *        60
PaMA_175194p0010   : ------------------------------------------------------------ :   -
PaMA_10428033p0010 : MNPIRPASRIGSIFKKEEQRLLIENDMKNYKSIDSTTSQDGLHPVHGSVGSSSDQALQKL :  60
AtSLAC1            : ----------------------------------MERKQSNAHSTFADINEVEDEAEQEL :  26

                               *        80         *       100         *       120
PaMA_175194p0010   : ------------------------------------------------------------ :   -
PaMA_10428033p0010 : HKVDELEEADYQENQHSMEISKQKLMSKLDKFESPRGAVDFEVKMKCSVLSVEEGKTKCL : 120
AtSLAC1            : QQQENNNNKRFSGNRG-PNRGKQRPFRGFSRQVSLETGFSVLNRE-----SRERDDKKSL :  80
                                                    ↑...CDPK6...↑               ↑.
                                                  ↑..OST1..↑

                              *       140         *       160         *       180
PaMA_175194p0010   : -------------------------MENQNSFTLEERSDGIYG--SGHR----------- :  22
PaMA_10428033p0010 : KQKSNPLEGKLGRQLSLGTGFSKWNKENGNSPKVLARSGASFSGFSSNKSKEMMKDQNYS : 180
AtSLAC1            : PRSGRSFGGFESGGIINGGDGRKTDFSMFRTKSTLSKQKSLLPSIIRERDIEN------- : 133
                     ..OST1...↑               ↑.......OST1........↑

                              *       200         *       220         *       240
PaMA_175194p0010   : ----DAGSVFLQS-----------------VSVEIPAARYFQALEGPELEVLRDCENVGL :  61
PaMA_10428033p0010 : LFGTKSGNALLQARKMEEQERQDHNIQQSRVHLNLPAGRYFDALKGPELDVLKDYEDT-L : 239
AtSLAC1            : SLRTEDGETKDDS-----------------INENVSAGRYFAALRGPELDEVKDNEDI-L : 175

                              *       260         *       280         *       300
PaMA_175194p0010   : PPVDKKWPFLLRFPNACFGMCIGLASQASLWKALSTSPAVKFLHIP-PQINLALWLLAVC : 120
PaMA_10428033p0010 : LPLDKKWPFLLRFPIGCFGICLGLGSQAILWKSLSEVYFMKFLQRPLVIINVVFWSLGLS : 299
AtSLAC1            : LPKEEQWPFLLRFPIGCFGICLGLSSQAVLWLALAKSPATNFLHIT-PLINLVVWLFSLV : 234
                           ↑.......... Motif1..........↑

                              *       320         *       340         *       360
PaMA_175194p0010   : IITMVFCTYFLKSIYHFEAVKREFCHPVRVNFFFAPWVASMTLAIAVPPVIA---KSVHP : 177
PaMA_10428033p0010 : ALIIIFITYSLKCMFYFEAVKREYYHPVRVNFFFAPWIICMFLSLGVPEAIT---EHIHP : 356
AtSLAC1            : VLVSVSFTYILKCIFYFEAVKREYFHPVRVNFFFAPWVVCMFLAISVPPMFSPNRKYLHP : 294
                                   ↑............Motif2..........↑

                              *       380         *       400         *       420
PaMA_175194p0010   : SVWCIFMAPLLILELKIYGQWLSGGEKRLSKVANPSTHLSVVGNFVGSLLAARLEWKEPA : 237
PaMA_10428033p0010 : ALWCIFMAPVFMLELKIYGQWLSGGKRRLSKVANPSSHLSVVGNFVGALLAARVKWKEPA : 416
AtSLAC1            : AIWCVFMGPYFFLELKIYGQWLSGGKRRLCKVANPSSHLSVVGNFVGAILASKVGWDEVA : 354
                                                           ↑......Motif3.....↑

                              *       440         *       460         *       480
PaMA_175194p0010   : KLLWAVGLAHYLVLFVTLYQRLPTSETLPKELHPVFFLFLAAPATASWAWETIFGEFDTV : 297
PaMA_10428033p0010 : KFFWAVGLGHYIVLFVTLYQRLPTSEALPQELHPVFFLFIAAPCTASVAWEAIYGDFDSV : 476
AtSLAC1            : KFLWAVGFAHYLVVFVTLYQRLPTSEALPKELHPVYSMFIAAPSAASIAWNTIYGQFDGC : 414
                                                ↑.........Motif4.......↑

                              *       500         *       520         *       540
PaMA_175194p0010   : SRIMYFVSLFIYTSLVVRIKLFRGFRFSISWWAYTYPMNSVSIATIRYSEQVEHLFTHVL : 357
PaMA_10428033p0010 : SRIAYFIALFLYTSLVVRINFFRGFRFSVAWWAYTFPMTAAAVATIRYSEAVRCTATKAL : 536
AtSLAC1            : SRTCFFIALFLYISLVARINFFTGFKFSVAWWSYTFPMTTASVATIKYAEAVPGYPSRAL : 474
                                               ↑........Motif5.......↑

                              *       560         *       580         *       600
PaMA_175194p0010   : AVLLSTVATITVFSIFVTTLLHAFVWKTLFPNDMAIAITTKQRNSINKSSPNPKLKVYDI : 417
PaMA_10428033p0010 : ALVLSIISFTTVLTLFVATILHAISG-SLFPNDLAIAITRKRYKSKALKENKKTAANSDF : 595
AtSLAC1            : ALTLSFISTAMVCVLFVSTLLHAFVWQTLFPNDLAIAITKRKLT----REKKPFKRAYDL : 530
                                                        ↑..OST1..↑

                              *       620         *       640         *       660
PaMA_175194p0010   : EQANTKPKGPVRTSFELLKATIRNHGQRREKPVYPDNILVDDKRHADEEQMLDRSASPKN : 477
PaMA_10428033p0010 : EQLHASP---IQQGMDMFTMKS----QQTEVAVHPDPLFNHKLP---------------- : 632
AtSLAC1            : KRWTKQA-------------------LAKKISAEKDFEAEEESHH--------------- : 556

PaMA_175194p0010   : AGHGPLD : 484
PaMA_10428033p001  : ------- :   -
AtSLAC1            : ------- :   -
```
