# Supplementary material for: Unique Responsiveness of Angiosperm Stomata to Elevated CO2 Explained by Calcium Signalling
Source: PLoS One. 2013 Nov 20;8(11):e82057. doi: 10.1371/journal.pone.0082057 (PMC3835710; doi:10.1371/journal.pone.0082057)
Supplement: Figure S6 — Lack of all key N-terminus OST1 phosphorylating domains found in angiosperms on the putative Selaginella SLAC1 proteins. Alignment of the Arabidopsis thaliana (At) SLAC1 protein sequence and the only four SLAC1-like proteins identified by BLAST searches of the genome of Selaginella moellendorffii (Sm) (Figure S4). Below the alignment is shown the Arabidopsis phosphorylation domain for AtCDPK6 in blue, while the putative phosphorylation domains for AtOST1 identified by Geiger et al. [43] (with positions refined to the conserved domains [59]) are shown in red. Yellow letters shaded in red indicate conserved phosphorylation sites in angiosperms [59], while the underlined phosphorylation domain for AtOST1 denotes the predominant phosphorylating domain in Arabidopsis [43]. Note the lack of conservation of all key N-terminus AtOST1 phosphorylating domains in all four identified SLAC1-like proteins of Selaginella. Transmembrane motifs for SLAC1 are shown in green [59]. Shading in the sequence alignment indicates the degree of amino acid conservation (black = 100%, dark grey = 80%, light grey = 60%). (PDF) [file pone.0082057.s006.pdf]

```
                    *        20         *        40         *        60
Sm53394  : ------------------------------------------------------------ :   -
Sm53336  : ------------------------------------------------------------ :   -
Sm122390 : ------------------------------------------------------------ :   -
Sm31887  : ------------------------------------------------------------ :   -
AtSLAC1  : MERKQSNAHSTFADINEVEDEAEQELQQQENNNNKRFSGNRGPNRGKQRPFRGFSRQVSL :  60
                                                                  ↑...
                                                                ↑.OST1

                    *        80         *       100         *       120
Sm53394  : ------------------------------EKYVLFRTRSSK-IEKQLSRLREEEIQQQQ :  29
Sm53336  : --------------------------------YNLFKTRSSKSLEKQIPWLSKQ------ :  22
Sm122390 : ------------------------------------------------------------ :   -
Sm31887  : ------------------------------------------------------------ :   -
AtSLAC1  : ETGFSVLNRESRERDDKKSLPRSGRSFGGFESGGIINGGDGRKTDFSMFRTKSTLSKQKS : 120
           CDPK6...↑            ↑...OST1...↑          ↑......OST1......
           ...↑

                    *       140         *       160         *       180
Sm53394  : QQHHQLDPGDHPEQQQQSSKPIHHEDHSAQVPAGRYFDALQGPELEQLKESEE-ALLPLD :  88
Sm53336  : -------NGDAPPPKFET---------DEELPAGRFFDALQGPELEVLKDSED-LLLPLD :  65
Sm122390 : --------MDEESALGTT--SNAP------VPTGRYFDALQGPELEVVESEQDLLLLPMD :  44
Sm31887  : AAAAAATAATTAAAVNGD--PTSPRPLEANVPAGRYLDALQGPELEVLKESED-LLLPTD :  57
AtSLAC1  : LLPSIIRERDIENSLRTEDGETKDDSINENVSAGRYFAALRGPELDEVKDNED-ILLPKE : 179
           ..↑

                    *       200         *       220         *       240
Sm53394  : QRWPFLLRFPISSFGVALGLGSQSILWKNLSTVPAMRFL-HAPEIINTVLWFLAAIALAG : 147
Sm53336  : QKWPFLLRVPANVFGVSIGLSAQSLLWAEIHDASSLSYLKHVPKGVLFALWSIGVFVHTV : 125
Sm122390 : KVWPFLLRFPVNSFGIVLGLGSQGILWKSLSSTPAMEEFVHIPKQVNLVLWCATLLAFVA : 104
Sm31887  : QTWPFLLRFPINSFGIVLGLGSQTILWKKLSLMPSMR-FLHIPLHINFVLWCVALCAYTL : 116
AtSLAC1  : EQWPFLLRFPIGCFGICLGLSSQAVLWLALAKSPATN-FLHITPLINLVVWLFSLVVLVS : 238
              ↑.......... Motif1..........↑

                    *       260         *       280         *       300
Sm53394  : IFTTYLFKAIFYLEAVRREYYHPVRANFFFAPWIAAMFLALGIPPSAGVT---LHPWIWC : 204
Sm53336  : LFSAYALKCVFYFEAVRREFHHPVRVNYFFAPWVAAMLLALSTPKDIAKR---LHPWLLG : 182
Sm122390 : ISLTYLLKCFLYFEAVRREFFHPVRINYFFAPCIGGMFLAIGLPDAVGLS---LHPSVWC : 161
Sm31887  : ISLTYVCKCIFYFEAVRREFYHPVRINFFFAPWVCGMFLTLGVPPTIAAS---IHPAAWL : 173
AtSLAC1  : VSFTYILKCIFYFEAVKREYFHPVRVNFFFAPWVVCMFLAISVPPMFSPNRKYLHPAIWC : 298
                         ↑...........Motif2..........↑

                    *       320         *       340         *       360
Sm53394  : CIMAPILALELKIYGQWLSGGQRRLSKVANPCTHLSVVGNFVGALLGATVGWIDGGIFFW : 264
Sm53336  : VFMGPVFALEVKIYGQWLFGGERRLSKVANPTTHLALTGNFVGSLLSHVVGWKEFAIFLF : 242
Sm122390 : ILMAPLFLLELKIYGQWLSGGDRRLSKVANPSTHLSIVGNFVGAVLGARVGWKEPAMFFW : 221
Sm31887  : VFMSPLVVLELKIYGQWLSGGERRLSKVANPATHVSIVGNFVGSLLGATVEWKELATFFW : 233
AtSLAC1  : VFMGPYFFLELKIYGQWLSGGKRRLCKVANPSSHLSVVGNFVGAILASKVGWDEVAKFLW : 358
                                       ↑......Motif3.....↑

                    *       380         *       400         *       420
Sm53394  : AIGLAHYLVLFVTLYQRLPTNEALPKELHPVFFLFIAAPSAASLAWEKITGDFGIVSRIA : 324
Sm53336  : AVGLAHYVVLFVTLYQRLPTNETLPKELHPVFFLFIAAPSIASEAWEMLACSFDYPARLI : 302
Sm122390 : AVGLAHYMVLFVTLYQRLPTNKRLPKDLHPVFFLFVAAPSTASVAWEDITGSFGYVSRIV : 281
Sm31887  : AVGLAHYVVVFVTLYQRLPTNELLTRDMHPVYFLFLAVPSTASVAWEKITSDFGYVSKLV : 293
AtSLAC1  : AVGFAHYLVVFVTLYQRLPTSEALPKELHPVYSMFIAAPSAASIAWNTIYGQFDGCSRTC : 418
                                ↑.........Motif4.......↑

                    *       440         *       460         *       480
Sm53394  : FFIALFLFSSL------IVRINFFRGFRFSLAWWAYTFPMTAISIASIHYCQEVSTPVTQ : 378
Sm53336  : HFVGLFLFISL------IVRLNFFRGFRFSIAWWAYTFPVAAIAMTANRYGLVVSSWLTD : 356
Sm122390 : YFVSLFLLLSL------VVRVNLFRGFRFSISWWAYTFPLGAAAIATIHYYSAVTCTLTR : 335
Sm31887  : FFLGLFLLFSLLRFYMQVVRVSIFRGFKFSLTWWAYTFPLTAVAIASIHYAVREPGLFTQ : 353
AtSLAC1  : FFIALFLYISL------VARINFFTGFKFSVAWWSYTFPMTTASVATIKYAEAVPGYPSR : 472
                                     ↑........Motif5.......↑

                    *       500         *       520         *       540
Sm53394  : ALATILSSVSSATVALLFVATMCHWLVWKTLFPNDLAIAITNRRRKRKR----------- : 427
Sm53336  : AMTSFFIFLSVAIVACVLVLSILHAVLWGTLLPNDMAIAITTHKKKK------------- : 403
Sm122390 : FLVVALSLASVLVVVSIFVTTLACACLYGTLFPNDQAIAITSEQHRKQHQLCKQQA---- : 391
Sm31887  : GLATVISFVAVTVVFLLFVFTLVHAFVWRTLFPNDIAIAITRKK---------------- : 397
AtSLAC1  : ALALTLSFISTAMVCVLFVSTLLHAFVWQTLFPNDLAIAITKRKLTREKKPFKRAYDLKR : 532
                                            ↑..OST1..↑

                    *       560
Sm53394  : ------------------------ :   -
Sm53336  : ------------------------ :   -
Sm122390 : ------------------------ :   -
Sm31887  : ------------------------ :   -
AtSLAC1  : WTKQALAKKISAEKDFEAEEESHH : 556
```
