# Supplementary material for: Unique Responsiveness of Angiosperm Stomata to Elevated CO2 Explained by Calcium Signalling
Source: PLoS One. 2013 Nov 20;8(11):e82057. doi: 10.1371/journal.pone.0082057 (PMC3835710; doi:10.1371/journal.pone.0082057)
Supplement: Table S1 — Experimental species including family and a brief description of the native habitat and ecology. (DOCX) [file pone.0082057.s007.docx]

**Table S1.** Experimental species including family and a brief description of the native habitat and ecology.

| **Species** | **Family** |  | **Habitat** |
| --- | --- | --- | --- |
| Angiosperms | | | |
| *Phoenix canariensis* Chabaud* | Arecaceae | Mediterranean palm | |
| *Senecio minimus* Poir.* | Asteraceae | Temperate ruderal herb | |
| *Lotus corniculatus* L.* | Fabaceae | Temperate grassland herb | |
| *Magnolia champaca* L.* | Magnoliaceae | Tropical rainforest tree | |
| *Brachychiton megaphyllus* Guymer* | Malvaceae | Tropical dry-forest shrub | |
| *Toona ciliata* M.Roem.* | Meliaceae | Tropical rainforest tree | |
| *Eucalyptus tenuiramis* Miq.* | Myrtaceae | Temperate dry-forest tree | |
| *Nothofagus cunninghamii* (Hook.) Oerst* | Nothofagaceae | Temperate rainforest tree | |
| *Epilobium ciliatum* Raf.* ^†^ | Onagraceae | Temperate ruderal herb | |
| *Triticum aestivum* L. (var. Machete) * | Poaceae | Domesticated cereal | |
| *Protea cynaroides* L. (cv. Little Prince)* | Proteaceae | Dry woodland shrub | |
| Gymnosperms | | | |
| *Agathis robusta* (C.Moore ex F.Muell.) Bailey* | Araucariaceae | Tropical rainforest canopy tree | |
| *Callitris macleayana* (F.Muell.) F.Muell.*^†^ | Cupressaceae | Subtropical forest tree | |
| *Cunninghamia lanceolata* (Lamb.) Hook.* | Cupressaceae | Temperate canopy tree | |
| *Metasequoia glyptostroboides* Hu and Cheng^†^ | Cupressaceae | Temperature deciduous tree | |
| *Cycas armstrongii* Miq.^†^ | Cycadaceae | Tropical dry-forest cycad | |
| *Ginkgo biloba* L.* | Ginkgoaceae | Temperate deciduous tree | |
| *Pinus caribaea* Morelet*^†^ | Pinaceae | Tropical dry forest tree | |
| *Acmopyle pancheri* Brongn. & Gris Pilg.* | Podocarpaceae | Tropical rainforest tree | |
| *Sciadopitys verticillata* (Thunb.) Siebold & Zucc. * | Sciadopityaceae | Temperate tree | |
| *Austrotaxus spicata* R.H.Compton* | Taxaceae | Tropical rainforest tree | |
| *Dioon edule* Lindl.* | Zamiaceae | Subtropical dry-forest cycad | |
| *Lepidozamia peroffskyana* Regal* | Zamiaceae | Subtropical wet-forest cycad | |
| Ferns and lycophytes | | | |
| *Asplenium scolopendrium* L.  (syn. *Phyllitis scolopendrium*) * | Aspleniaceae | Temperate epiphyte | |
| *Cyathea cunninghamii* Hook.f. * | Cyatheaceae | Temperate tree-fern | |
| *Pteridium esculentum* (G. Forst.) Cockayne* | Dennstaedtiaceae | Temperate open forest pioneer | |
| *Equisetum hyemale* L.* | Equisetaceae | Temperate swamp horsetail | |
| *Dicranopteris linearis* (Burm.f.) Underw.* | Gleicheniaceae | Tropical rhizomatous pioneer | |
| *Lygodium flexuosum* (Linn.) Sw.* | Lygodiaceae | Tropical climber | |
| *Marsilea hirsuta* R. Br.* | Marsileaceae | Subtropical amphibious fern | |
| *Todea barbara* (L.) T.Moore* | Osmundaceae | Temperate wet-forest tree-fern | |
| *Pyrrosia lingua* (Thunb.) Farw. * | Polypodiaceae | Subtropical canopy epiphyte | |
| *Psilotum nudum* (L.) Beauv. * | Psilotaceae | Tropical and temperate epiphyte | |
| *Ceratopteris richardii* Brongn. (line Hnn) ^†^ | Pteridaceae | Tropical aquatic | |
| *Huperzia phlegmarioides* (Gaudich.) Rothm. * | Lycopodiaceae | Tropical epiphyte | |
| *Selaginella uncinata* (Desv.) Spring*^†^ | Selaginellaceae | Tropical understorey creeper | |

*Species used in both light and dark CO_2_ gas exchange experiments

^†^Species used in stomatal aperture experiments
