# Supplementary material for: Unique Responsiveness of Angiosperm Stomata to Elevated CO2 Explained by Calcium Signalling
Source: PLoS One. 2013 Nov 20;8(11):e82057. doi: 10.1371/journal.pone.0082057 (PMC3835710; doi:10.1371/journal.pone.0082057)
Supplement: Table S2 — Photosynthetic rates (µmol m-2 s-1) and internal leaf CO2 concentration (Ci) at current ambient atmospheric CO2 concentration (400 µmol mol-1) and the ratio of Ci to Ca at low CO2 (100 µmol mol-1) in the dark for each experimental species. (DOCX) [file pone.0082057.s008.docx]

**Table S2.** Photosynthetic rates (µmol m^-2^ s^-1^) and internal leaf CO_2_ concentration (*C_i_*) at current ambient atmospheric CO_2_ concentration (400 µmol mol^-1^) and the ratio of *C_i_* to *C_a_* at low CO_2_ (100 µmol mol^-1^) in the dark for each experimental species.

| **Species** | **Photosynthetic rate** |  | **Internal leaf CO_2_ in the light**  **(400 µmol mol^-1^)** | ***C_i_*:*C_a_* at low CO_2_ in the dark** |
| --- | --- | --- | --- | --- |
| Angiosperms | | | | |
| *Phoenix canariensis* | 7.4 | 264 | | 1.00 |
| *Senecio minimus* | 15.2 | 280 | | 1.10 |
| *Lotus corniculatus* | 14.7 | 251 | | 1.05 |
| *Magnolia champaca* | 8.1 | 260 | | 1.29 |
| *Brachychiton megaphyllus* | 17.0 | 368 | | 1.02 |
| *Toona ciliata* | 6.9 | 249 | | 1.00 |
| *Eucalyptus tenuiramis* | 19.7 | 321 | | 1.00 |
| *Nothofagus cunninghamii* | 7.5 | 229 | | 1.11 |
| *Epilobium ciliatum* | 6.2 | 307 | | 1.01 |
| *Triticum aestivum* var. Machete | 22.0 | 306 | | 1.01 |
| *Protea cynaroides* cv. Little Prince | 9.5 | 262 | | 1.03 |
| Gymnosperms | | | | |
| *Agathis robusta* | 4.7 | 325 | | 1.10 |
| *Callitris macleayana* | 9.3 | 285 | | 1.31 |
| *Cunninghamia lanceolata* | 4.5 | 307 | | 1.15 |
| *Ginkgo biloba* | 4.6 | 297 | | 1.42 |
| *Pinus caribaea* | 13.0 | 298 | | 1.00 |
| *Acmopyle pancheri* | 4.6 | 233 | | 1.17 |
| *Sciadopitys verticillata* | 5.5 | 283 | | 1.14 |
| *Austrotaxus spicata* | 5.3 | 274 | | 1.08 |
| *Dioon edule* | 7.7 | 279 | | 1.18 |
| *Lepidozamia peroffskyana* | 5.0 | 222 | | 1.23 |
| Ferns and lycophytes | | | | |
| *Asplenium scolopendrium* | 3.3 | 284 | | 1.30 |
| *Cyathea cunninghamii* | 3.5 | 257 | | 1.07 |
| *Pteridium esculentum* | 12.5 | 276 | | 1.05 |
| *Equisetum hyemale* | 4.0 | 315 | | 1.00 |
| *Dicranopteris linearis* | 5.8 | 270 | | 1.07 |
| *Lygodium flexuosum* | 2.6 | 346 | | 1.05 |
| *Marsilea hirsuta* | 5.0 | 289 | | 1.01 |
| *Todea barbara* | 4.6 | 320 | | 1.07 |
| *Pyrrosia lingua* | 3.9 | 251 | | 1.16 |
| *Psilotum nudum* | 3.4 | 301 | | 1.11 |
| *Huperzia phlegmarioides* | 2.9 | 269 | | 1.08 |
| *Selaginella uncinata* | 3.5 | 304 | | 1.12 |
