# Supplementary material for: Unique Responsiveness of Angiosperm Stomata to Elevated CO2 Explained by Calcium Signalling
Source: PLoS One. 2013 Nov 20;8(11):e82057. doi: 10.1371/journal.pone.0082057 (PMC3835710; doi:10.1371/journal.pone.0082057)
Supplement: Table S3 — Accession numbers and gene models of CDPKs and related protein kinase sequences in the conifer Picea abies (Pa) (including details on expressed tissue) lycophyte Selaginella moellendorffii (Sm) and angiosperms Arabidopsis thaliana (At), Oryza sativa (Os) and Mesembryanthemum crystallinum (Mc) used in the alignment and subsequent phylogram in Figure S1. Predicted amino acid sequence gene models and information on expressed tissue was identified in BLAST searches of the P. abies (http://congenie.org/) and S. moellendorffii (http://www.phytozome.net) genomes. Angiosperm Genbank accession numbers are given for each of the protein sequences used in the alignment and phylogenetic neighbour-joining tree. (DOCX) [file pone.0082057.s009.docx]

**Table S3.** Accession numbers and gene models of CDPKs and related protein kinase sequences in the conifer *Picea abies* (Pa) (including details on expressed tissue) lycophyte *Selaginella moellendorffii* (Sm) and angiosperms *Arabidopsis thaliana* (At), *Oryza sativa* (Os) and *Mesembryanthemum crystallinum* (Mc) used in the alignment and subsequent phylogram in Figure S1. Predicted amino acid sequence gene models and information on expressed tissue was identified in BLAST searches of the *P. abies* (<http://congenie.org/>) and *S. moellendorffii* (<http://www.phytozome.net>) genomes. Angiosperm Genbank accession numbers are given for each of the protein sequences used in the alignment and phylogenetic neighbour-joining tree.

| **Gene** | **Gene model/accession #** | **Expressed tissue** |
| --- | --- | --- |
| PaCDPK1 | MA­_10431349g0010 | Everywhere |
| PaCDPK2 | MA_9458g0010 | Male cones |
| PaCDPK3 | MA_85910g0010 | Everywhere |
| PaCDPK4 | MA_8898899g0010 | Female cones |
| PaCDPK5 | MA_110181g0010 | Female cones |
| PaCDPK6 | MA_115550g0010 | Leaves |
| PaCDPK7 | MA_10429609g0010 | Everywhere |
| PaCDPK8 | MA_18543g0010 | Wood |
| PaCDPK9 | MA_98632g0020 | Wood |
| PaCDPK10 | MA_10437101g0020 | Wood and leaves |
| PaCDPK11 | MA_10435930g0050 | Wood |
| PaCDPK12 | MA_10435930g0030 | Wood and female cones |
| PaCDPK13 | MA_13110g0010 | Buds and male cones |
| PaCDPK14 | MA_57791g0010 | Buds and leaves |
| PaCDPK15 | MA_10437101g0010 | Wood and leaves |
| PaCRK1 | MA_17088g0010 |  |
| PaCRK2 | MA_3361g0010 |  |
| PaPEPRK1 | MA_130508g0010 |  |
| PaPEPRK2 | MA_10429985g0010 |  |
| PaPEPRK3 | MA_10432713g0010 |  |
| PaPEPRK4 | MA_502954g0010 |  |
| PaPEPRK5 | MA_8587931g0010 |  |
| PaPPCK1 | MA_48600g0010 |  |
| PaSNRK | MA_146450g0010 |  |
| SmCDPK1 | 92726 |  |
| SmCDPK2 | 164119 |  |
| SmCDPK3 | 105020 |  |
| SmCDPK4 | 105846 |  |
| SmCDPK5 | 165073 |  |
| SmCDPK6 | 99178 |  |
| SmCDPK7 | 152133 |  |
| SmCDPK8 | 118877 |  |
| SmCDPK9 | 96034 |  |
| SmCDPK10 | 231127 |  |
| SmCDPK11 | 122526 |  |
| SmCRK1 | 73433 |  |
| SmCRK2 | 173444 |  |
| SmPEPRK | 99940 |  |
| SmSNRK1.1 | 233667 |  |
| SmSNRK1.2 | 80443 |  |
| SmSNRK3.1 | 234951 |  |
| AtCDPK1 | At5g04870 |  |
| AtCDPK2 | At3g10660 |  |
| AtCDPK3 | At4g23650 |  |
| AtCDPK4 | At4g09570 |  |
| AtCDPK5 | At4g35310 |  |
| AtCDPK6 | At2g17290 |  |
| AtCDPK7 | At5g12150 |  |
| AtCDPK8 | At5g19450 |  |
| AtCDPK9 | At3g20410 |  |
| AtCDPK10 | At1g18890 |  |
| AtCDPK11 | At1g35670 |  |
| **Gene** | **Gene model/accession #** | **Expressed tissue** |
| AtCDPK12 | AAA67657 |  |
| AtCDPK13 | At3g51850 |  |
| AtCDPK14 | At2g41860 |  |
| AtCDPK15 | At4g21940 |  |
| AtCDPK16 | At2g17890 |  |
| AtCDPK17 | At5g12180 |  |
| AtCDPK18 | At4g36070 |  |
| AtCDPK19 | At1g61950 |  |
| AtCDPK20 | At2g38910 |  |
| AtCDPK21 | At4g04720 |  |
| AtCDPK22 | At4g04710 |  |
| AtCDPK23 | At4g04740 |  |
| AtCDPK24 | At2g31500 |  |
| AtCDPK25 | At2g35890 |  |
| AtCDPK26 | At4g38230 |  |
| AtCDPK27 | At4g04700 |  |
| AtCDPK28 | At5g66210 |  |
| AtCDPK29 | At1g76040 |  |
| AtCDPK30 | At1g74740 |  |
| AtCDPK31 | 5732059 |  |
| AtCDPK32 | At3g57530 |  |
| AtCDPK33 | At1g50700 |  |
| AtCDPK34 | At5g19360 |  |
| AtCRK1 | At2g41140 |  |
| AtCRK6 | At3g49370 |  |
| AtPEPRK1 | At1g12580 |  |
| AtPEPRK2 | At1g12880 |  |
| AtPPCK1 | At1g08650 |  |
| AtPPCK2 | At3g04530 |  |
| AtSNRK1.1 | At3g01090 |  |
| AtSNRK2.5 | At5g63650 |  |
| AtSNRK3.9 | At4g18700 |  |
| OsPPCK | 6625818 |  |
| McPPCK | 6175636 |  |
