# Supplementary material for: Unique Responsiveness of Angiosperm Stomata to Elevated CO2 Explained by Calcium Signalling
Source: PLoS One. 2013 Nov 20;8(11):e82057. doi: 10.1371/journal.pone.0082057 (PMC3835710; doi:10.1371/journal.pone.0082057)
Supplement: Table S4 — Sequence information including species, gene model and source for the numbered protein sequences used to construct the phylogenetic neighbour-joining tree shown in Figure S4. (DOCX) [file pone.0082057.s010.docx]

**Table S4.** Sequence information including species, gene model and source for the numbered protein sequences used to construct the phylogenetic neighbour-joining tree shown in Figure S4.

| **No.** | **Species** | **Gene model** | **Source** |
| --- | --- | --- | --- |
| 1 | *Chlamydomonas reinhardtii* | Cre13.g584200.t1.2 | http://www.jgi.doe.gov/ |
| 2 | *Chlorella variabilis* | ChlNC64A_153657 | http://www.jgi.doe.gov/ |
| 3 | *Chlorella variabilis* | ChlNC64A_1_135500 | http://www.jgi.doe.gov/ |
| 4 | *Asterochloris* sp. | Astpho1_23384 | http://www.jgi.doe.gov/ |
| 5 | *Coccomyxa subellipsoidea* | 41988 | http://www.jgi.doe.gov/ |
| 6 | *Chlorella variabilis* | ChlNC64A_1_23135 | http://www.jgi.doe.gov/ |
| 7 | *Sorghum bicolor* | Sb03g000220.1 | http://www.phytozome.net/ |
| 8 | *Sorghum bicolor* | Sb09g002500.1 | http://www.phytozome.net/ |
| 9 | *Sorghum bicolor* | Sb03g028360.1 | http://www.phytozome.net/ |
| 10 | *Sorghum bicolor* | Sb09g029920.1 | http://www.phytozome.net/ |
| 11 | *Sorghum bicolor* | Sb09g029930.1 | http://www.phytozome.net/ |
| 12 | *Sorghum bicolor* | Sb09g029910.1 | http://www.phytozome.net/ |
| 13 | *Sorghum bicolor* | Sb07g003180.1 | http://www.phytozome.net/ |
| 14 | *Oryza sativa* | 01g28840.1 | http://www.phytozome.net/ |
| 15 | *Oryza sativa* | 05g13320.1 | http://www.phytozome.net/ |
| 16 | *Oryza sativa* | 07g08350.1 | http://www.phytozome.net/ |
| 17 | *Oryza sativa* | 01g43460.2 | http://www.phytozome.net/ |
| 18 | *Oryza sativa* | 04g48530.1 | http://www.phytozome.net/ |
| 19 | *Oryza sativa* | 05g18670.1 | http://www.phytozome.net/ |
| 20 | *Solanum lycopersicum* | Solyc08g079770.2.1 | http://www.phytozome.net/ |
| 21 | *Solanum lycopersicum* | Solyc03g007770.2.1 | http://www.phytozome.net/ |
| 22 | *Solanum lycopersicum* | Solyc06g036440.1.1 | http://www.phytozome.net/ |
| 23 | *Solanum lycopersicum* | Solyc09g014610.2.1 | http://www.phytozome.net/ |
| 24 | *Solanum lycopersicum* | Solyc07g051950.2.1 | http://www.phytozome.net/ |
| 25 | *Solanum lycopersicum* | Solyc04g080990.1.1 | http://www.phytozome.net/ |
| 26 | *Solanum lycopersicum* | Solyc03g031590.2.1 | http://www.phytozome.net/ |
| 27 | *Malus domestica* | MDP0000413940 | http://www.phytozome.net/ |
| 28 | *Sorghum bicolor* | Sb09g007180.1 | http://www.phytozome.net/ |
| 29 | *Ricinus communis* | 30169.m006568 | http://www.phytozome.net/ |
| 30 | *Sorghum bicolor* | Sb06g026050.1 | http://www.phytozome.net/ |
| 31 | *Malus domestica* | MDP0000841464 | http://www.phytozome.net/ |
| 32 | *Malus domestica* | MDP0000898762 | http://www.phytozome.net/ |
| 33 | *Malus domestica* | MDP0000234689 | http://www.phytozome.net/ |
| 34 | *Glycine max* | Glyma10g37370.1 | http://www.plantgdb.org/GmGDB/ |
| 35 | *Phaseolus vulgaris* | Phvul.007G076700.1 | http://www.phytozome.net/ |
| 36 | *Phaseolus vulgaris* | Phvul.004G102800.1 | http://www.phytozome.net/ |
| 37 | *Phaseolus vulgaris* | Phvul.003G080700.1 | http://www.phytozome.net/ |
| 38 | *Phaseolus vulgaris* | Phvul.011G102100.1 | http://www.phytozome.net/ |
| 39 | *Phaseolus vulgaris* | Phvul.009G013800.1 | http://www.phytozome.net/ |
| 40 | *Vitis vinifera* | GSVIVT01019915001 | http://www.phytozome.net/ |
| 41 | *Vitis vinifera* | GSVIVT01028789001 | http://www.phytozome.net/ |
| 42 | *Vitis vinifera* | GSVIVT01009814001 | http://www.phytozome.net/ |
| 43 | *Zea mays* | GRMZM2G106921_T01 | http://www.phytozome.net/ |
| 44 | *Zea mays* | GRMZM2G061469_T01 | http://www.phytozome.net/ |
| 45 | *Zea mays* | GRMZM2G447657_T01 | http://www.phytozome.net/ |
| 46 | *Zea mays* | GRMZM2G080887_T01 | http://www.phytozome.net/ |
| 47 | *Zea mays* | GRMZM2G518198_T01 | http://www.phytozome.net/ |
| 48 | *Zea mays* | GRMZM2G074540_T01 | http://www.phytozome.net/ |
| 49 | *Brachypodium distachyon* | Bradi2g08670.1 | http://www.phytozome.net/ |
| 50 | *Eucalyptus grandis* | Eucgr.D01960.1 | http://www.phytozome.net/ |
| 51 | *Eucalyptus grandis* | Eucgr.K00304.1 | http://www.phytozome.net/ |
| 52 | *Eucalyptus grandis* | Eucgr.K00789.1 | http://www.phytozome.net/ |
| 53 | *Eucalyptus grandis* | Eucgr.E04014.1 | http://www.phytozome.net/ |
| 54 | *Eucalyptus grandis* | Eucgr.E04018.1 | http://www.phytozome.net/ |
| 55 | *Eucalyptus grandis* | Eucgr.E04013.1 | http://www.phytozome.net/ |
| 56 | *Linum usitatissimum* | Lus10006678 | http://www.phytozome.net/ |
| 57 | *Linum usitatissimum* | Lus10027553 | http://www.phytozome.net/ |
| 58 | *Linum usitatissimum* | Lus10015133 | http://www.phytozome.net/ |
| 59 | *Arabidopsis lyrata* | 471398 | http://www.phytozome.net/ |
| 60 | *Arabidopsis lyrata* | 354000 | http://www.phytozome.net/ |
| 61 | *Arabidopsis lyrata* | 489261 | http://www.phytozome.net/ |
| 62 | *Arabidopsis lyrata* | 338156 | http://www.phytozome.net/ |
| 63 | *Arabidopsis lyrata* | 893261 | http://www.phytozome.net/ |
| 64 | *Capsella rubella* | Carubv10011981m | http://www.phytozome.net/ |
| 65 | *Capsella rubella* | Carubv10006291m | http://www.phytozome.net/ |
| **No.** | **Species** | **Gene model** | **Source** |
| 66 | *Capsella rubella* | Carubv10000447m | http://www.phytozome.net/ |
| 67 | *Capsella rubella* | Carubv10020508m | http://www.phytozome.net/ |
| 68 | *Capsella rubella* | Carubv10021993m | http://www.phytozome.net/ |
| 69 | *Thellungiella halophila* | Thhalv10007262m | http://www.phytozome.net/ |
| 70 | *Thellungiella halophila* | Thhalv10024941m | http://www.phytozome.net/ |
| 71 | *Thellungiella halophila* | Thhalv10003801m | http://www.phytozome.net/ |
| 72 | *Thellungiella halophila* | Thhalv10023540m | http://www.phytozome.net/ |
| 73 | *Thellungiella halophila* | Thhalv10023519m | http://www.phytozome.net/ |
| 74 | *Brassica rapa* | Bra019770 | http://www.phytozome.net/ |
| 75 | *Brassica rapa* | Bra010388 | http://www.phytozome.net/ |
| 76 | *Brassica rapa* | Bra040840 | http://www.phytozome.net/ |
| 77 | *Brassica rapa* | Bra026294 | http://www.phytozome.net/ |
| 78 | *Brassica rapa* | Bra009724 | http://www.phytozome.net/ |
| 79 | *Brassica rapa* | Bra026471 | http://www.phytozome.net/ |
| 80 | *Brassica rapa* | Bra029390 | http://www.phytozome.net/ |
| 81 | *Brassica rapa* | Bra027058 | http://www.phytozome.net/ |
| 82 | *Carica papaya* | evm.model.supercontig_29.54 | http://www.phytozome.net/ |
| 83 | *Carica papaya* | evm.model.supercontig_3.11 | http://www.phytozome.net/ |
| 84 | *Carica papaya* | evm.model.supercontig_87.98 | http://www.phytozome.net/ |
| 85 | *Citrus clementina* | Ciclev10019450m | http://www.phytozome.net/ |
| 86 | *Citrus clementina* | Ciclev10006633m | http://www.phytozome.net/ |
| 87 | *Citrus clementina* | Ciclev10017595m | http://www.phytozome.net/ |
| 88 | *Citrus clementina* | clev10003716m | http://www.phytozome.net/ |
| 89 | *Ricinus communis* | 30190.m011063 | http://www.phytozome.net/ |
